# Network-based identification of biomarkers for colon adenocarcinoma

**DOI:** 10.1186/s12885-020-07157-w

**Published:** 2020-07-17

**Authors:** Fuyan Hu, Qing Wang, Zhiyuan Yang, Zeng Zhang, Xiaoping Liu

**Affiliations:** 1 Department of Statistics, School of Science, Wuhan University of Technology, 122 Luoshi Road, Wuhan, China; 2 Department of Traditional Chinese Medicine of Wuhan Puren Hospital, Affiliated Hospital of Wuhan University of Science and Technology, Benxi Street 1#, Qingshan District, Wuhan, Hubei P.R. China; 3 College of Life Information Science & Instrument Engineering, Hangzhou Dianzi University, Hangzhou, People’s Republic of China; 4 School of Mathematics and Statistics, Shandong University, Weihai, 264209 China

**Keywords:** Sample-specific network, Functional network, Diagnostic biomarkers, Prognosis biomarkers, Subtyping

## Abstract

**Background:**

As one of the most common cancers with high mortality in the world, we are still facing a huge challenge in the prevention and treatment of colon cancer. With the rapid development of high throughput technologies, new biomarkers identification for colon cancer has been confronted with the new opportunities and challenges.

**Methods:**

We firstly constructed functional networks for each sample of colon adenocarcinoma (COAD) by using a sample-specific network (SSN) method which can construct individual-specific networks based on gene expression profiles of a single sample. The functional genes and interactions were identified from the functional networks, respectively.

**Results:**

Classification and subtyping were used to test the function of the functional genes and interactions. The results of classification showed that the functional genes could be used as diagnostic biomarkers. The subtypes displayed different mechanisms, which were shown by the functional and pathway enrichment analysis for the representative genes of each subtype. Besides, subtype-specific molecular patterns were also detected, such as subtype-specific clinical and mutation features. Finally, 12 functional genes and 13 functional edges could serve as prognosis biomarkers since they were associated with the survival rate of COAD.

**Conclusions:**

In conclusion, the functional genes and interactions in the constructed functional network could be used as new biomarkers for COAD.

## Background

Colorectal cancer (CRC), which has a poor prognosis and a high mortality rate, is the most common gastrointestinal malignancy and the second major cause of deaths related to cancer in the world [1]. Overall CRC incidence and death rates have been declining over the past decades due to the advances in medicine, such as screening colonoscopy, radiotherapy, adjuvant and neoadjuvant therapy, and targeted therapies [2]. Despite that, approximately half of CRC patients treated with surgical resection recurred and died within 5 years [3]. Colon adenocarcinoma (COAD) is one of the major types of CRC.

With the development of high-throughput sequencing technologies, it was not only used on many crucial genetic and epigenetic alternations discovered for cancers, but also identified meaningful cancer biomarkers for diagnosis, prognosis and treatment prediction [4–9]. Biomarkers which can serve as diagnostic factors, prognostic indicators and drug targets for targeted therapy may bring a breakthrough in improving the prevention and treatment of CRC [10, 11]. However, most of the existing biomarkers have not been applied successfully in clinic. Many existing biomarkers focused on the genes with significant differential expression and the genes without significantly differential expression. However, gene expression is usually unstable, and it can change with state and environment. Network biomarkers usually identify gene modules from a molecular network and focus on the overall change of gene module from normal to disease on system level. So network-based biomarkers are better than single molecules [12, 13], and it can avoid the unstable factors for gene expression of a single gene and improve the stability of results.

In this study, we identified novel biomarkers by constructing a functional network for colon adenocarcinoma (COAD) based on sample-specific network (SSN) method [14] to avoid the disadvantages of single gene biomarkers. The results showed that our biomarkers could be used as diagnosis and prognosis biomarkers for COAD. What is more, we classified COAD into six subtypes using the gene expression profile of the biomarker genes.

To figure out the different mechanisms of each subtype, the representative genes were identified for each subtype; the enrichment analysis was done to gene ontology (GO) and Kyoto Encyclopedia of Genes and Genomes (KEGG, https://www.kegg.jp) pathway; and the associations among the subtypes, clinical and somatic alteration features were also assessed. In the end, different biomarkers were suggested for the precision medicine of COAD.

## Methods

### Datasets and networks

Multiplatform genomics datasets included gene expression profiles and somatic mutation in MAF (Mutation Annotation Format) files were downloaded from The Cancer Genome Atlas (TCGA, http://cancergenome.nih.gov/). And the clinical data were obtained through the TCGA Data Commons (https://gdc.cancer.gov/). Two other validation datasets were downloaded from Gene Expression Omnibus (GEO, https://www.ncbi.nlm.nih.gov/gds) with accession number GSE21510 and GSE39582. Besides, it was used as a background network that the functional association network with high confidence (experiment lab score > 300) which includes direct (physical) and indirect (functional) associations obtaining from the STRING database (version 10.5, http://string-db.org) [15].

### Visualizing and summary of mutation datasets

The mutations from MAF files were visualized and summarized through summary plots and oncoplots using the R/Bioconductor maftools package [16].

### Constructing SSN (sample-specific Network) for each sample

An SSN for each sample was constructed by a sample-specific network (SSN) method [14], which can infer individual-specific networks based on the expression data of a single sample from the following strategies. Firstly, the normal samples were considered as reference samples and a reference network was obtained by computing Pearson correlation coefficient (*PCC*) of each pair of molecules as an edge in the background network, which was conducted from STRING protein-protein interaction (PPI) network. And then, a perturbed network was constructed by adding a single sample to the reference samples and computing *PCC*s again. Finally, edges were kept to construct an SSN for this single sample if they showed statistically significant differential *PCC*s (*ΔPCC*s) based on the evaluation of SSN theory when comparing the perturbed network with the reference network.

### Functional network identification for cancer

Specific SSN for each tumor sample was obtained by deleting the edges presented in normal samples. If an edge appeared in more than 90% SSNs of tumor samples, the edge would be collected to form a functional network for COAD. The nodes and edges in the functional network were used as representative features for COAD, which were named as functional genes and functional interactions of COAD, respectively.

### The enrichment analysis of GO and KEGG pathway

The enrichment analysis of GO and KEGG pathway for functional genes were performed using DAVID web service (https://david.ncifcrf.gov/) [17, 18] with specifying a *p*-value< 0.05 for statistical significance.

Furthermore, genes in five known cancer gene sets were used as a proxy for the potential cancer-related genes including the curated gene sets in pathway in cancer (hsa05200), colorectal cancer (hsa05210), cancer gene census [19], pan-caner driver genes [20], and cancer driver genes [21]. And the probability *p*-values that can reflect whether functional genes are significantly enriched in these known cancer gene sets were calculated by the following formula [22]:

1 $$ p- value=1-\sum \limits_{i=0}^{k-1}\frac{\left(\begin{array}{c}A\\ {}i\end{array}\right)\left(\begin{array}{c}N-A\\ {}n-i\end{array}\right)}{\left(\begin{array}{c}N\\ {}n\end{array}\right)}, $$

where *N* is the total number of genes of the human genome, *A* is the number of genes in a known cancer gene set, *n* is the number of functional genes, *k* is the number of overlapping genes between functional genes and the known cancer gene set. If the *p*-value is less than 0.05, then it means that the functional genes are significantly enriched in the known cancer gene set. And then Venn diagrams were used to display the relationship between functional genes and the five known cancer gene sets.

### Functional genes as diagnostic biomarkers

To check whether functional genes can be used as diagnostic biomarkers for colon adenocarcinoma, 5-fold cross-validation was conducted to perform normal/tumor classification by a support vector machine (SVM), which was implemented in R with function ‘ksvm’ in ‘kernlab’ package. And the receiver operating characteristic (ROC) curve was drawn by R using the ‘ROCR’ package. In detail, TCGA data were used as training and test set, and GSE21510 data were used as an independent external validation dataset. To settle the problem of data imbalance, TCGA tumor data were divided into subgroups to make sure each subgroup had almost the same sample size with TCGA normal dataset. And then SVM model with 5-fold cross-validation was performed for each tumor subgroup and normal samples. Furthermore, hierarchical clustering was performed by using the gene expression of functional genes in both tumor and normal samples. And then heat maps were used to show the results.

### Colon adenocarcinoma subtypes and survival analysis

Colon adenocarcinoma samples were divided into subtypes by consensus clustering algorithm [23] using the expression data of functional genes. Consensus clustering was performed by ConsensusClusterPlus R-package using 1000 iterations, 80% sample resampling from 2 to 10 clusters, Ward linkage and the distance of Pearson correlation coefficient. Then one clustering solution was selected as a subtype solution. Differentially expressed genes (DEGs) associated with each subtype were identified by carrying out a two-sided *t*-test for each gene by comparing this subtype with the rest subtypes, and then the unique top 100 upregulated DEGs and downregulated DEGs with the lowest *p*-value were selected as representative genes for each subtype. Then their enriched biological processes and KEGG pathways were compared using the R package ‘clusterProfiler’ [24] which can compare biological themes among gene clusters. Subtype-specific clinical features and somatic alteration features were also assessed. Besides, Kaplan-Meier survival curves were drawn for subtypes and log-rank *p*-values were computed using the R package ‘survival’ [25].

### Prognostic prediction of COAD using functional genes and interactions

Association of functional genes and interactions with patients’ overall survival were assessed by Kaplan-Meier survival curves and the log-rank tests. Based on the expression level of functional genes or *ΔPCC*s of interactions, samples were divided into two subgroups with low- and high- expression. And then a univariate Cox regression analysis was done for each functional gene and interaction. Furthermore, a multivariate Cox regression analysis was further done to investigate and control the influences of the confounders on functional genes or interactions with *p*-value less than 0.05 in the univariate Cox regression analysis. The confounders included sex information, pathologic stages, retrospective collection indicator, race, the year of initial pathologic diagnosis, age at initial pathologic diagnosis and microsatellite status. Functional genes and interactions were identified as prognosis biomarkers for cancer when they showed significant differences between the low- and high- expression subgroups in both univariate and multivariate cox analysis. The function ‘coxph’ in R was used to do this job.

## Results

### Summary of datasets

There were 20,501 genes in 454 tumor and 41 normal samples for the dataset of gene expression of COAD in TCGA. The genes were removed if they did not express in more than 50% samples, and then 17,914 genes were left for further study. The first validation set (GSE21510) included 123 tumor samples and 25 normal samples. The second validation set (GSE39582) included 585 tumors among which 579 tumors had survival information and 19 patients had adjacent nontumor tissues.

The clinical data were matched to the gene expression profile. Among the 454 patients, 450 patients had clinical information with 395 patients alive and 45 patients dead. However, due to missing data of our selected confounders, only 258 samples were kept for the multivariate Cox regression analysis. The overall information of the 258 patients was listed in Table S1.

The summary of the mutation was drawn by maftools (Figure S1). There were 9 types of mutations in the MAF file. The number distribution for each type of mutation was shown by a bar plot and the one with the maximum frequency was Missense_Mutation; SNP was the most common variant type; the most common SNV type was C > T; variants per sample distribution were presented by a stacked barplot; variant types were displayed as a boxplot summarized by Variant_Classification; the top 10 genes (*TTN*, *APC*, *MUC16*, *SYNE1*, *TP53*, *FAT4*, *KRAS*, *RYR2*, *PIK3CA*, and *ZFHX4*) with the most mutations were shown by a stacked barplot (Figure S1).

STRING PPI data with lab score > 300 includes 15,436 genes and 217,626 interactions. After mapping 17,914 genes with expression information on PPI, a background network was constructed for the study, which involved 13,235 genes and 164,115 interactions.

### SSN analysis reveals a functional network for cancer

**Fig. 1:**
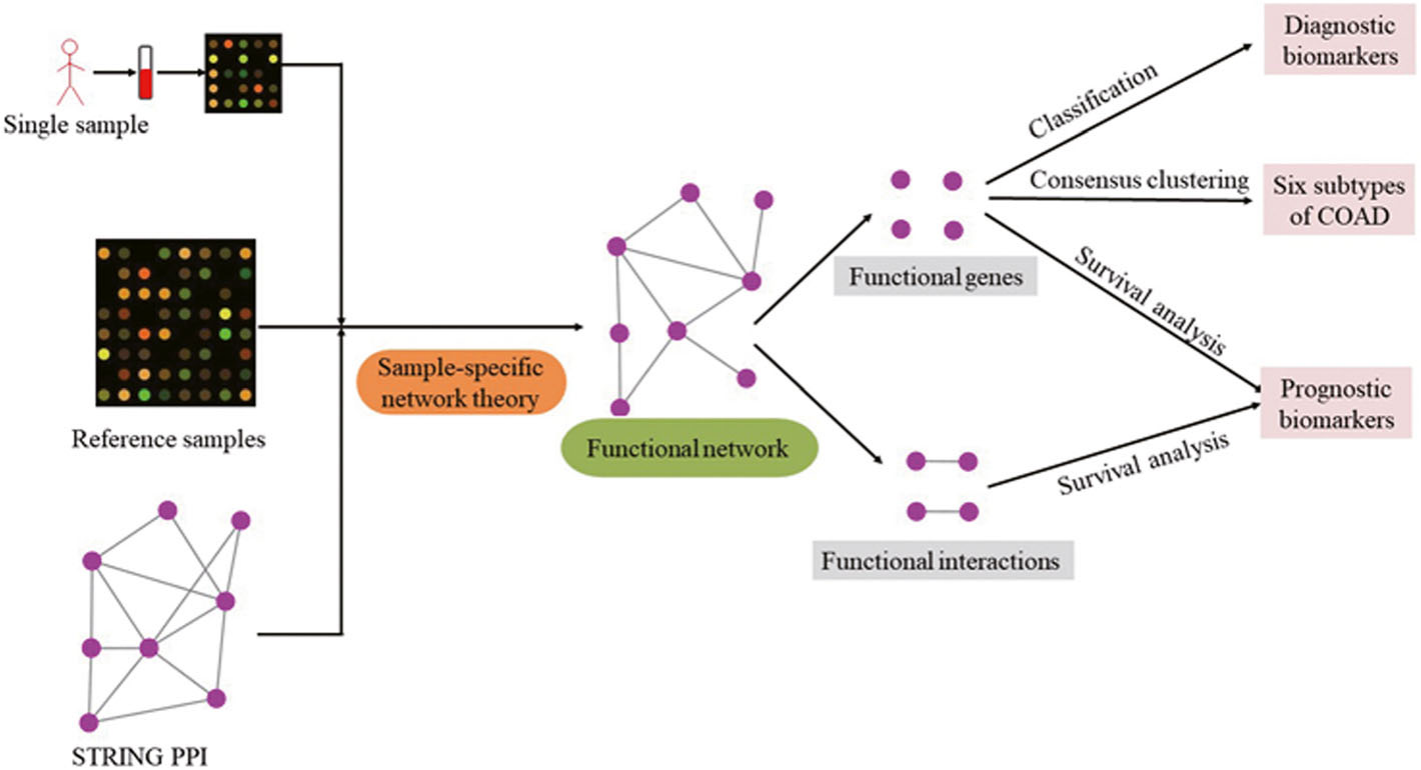
The pipeline of our method

**Fig. 2:**
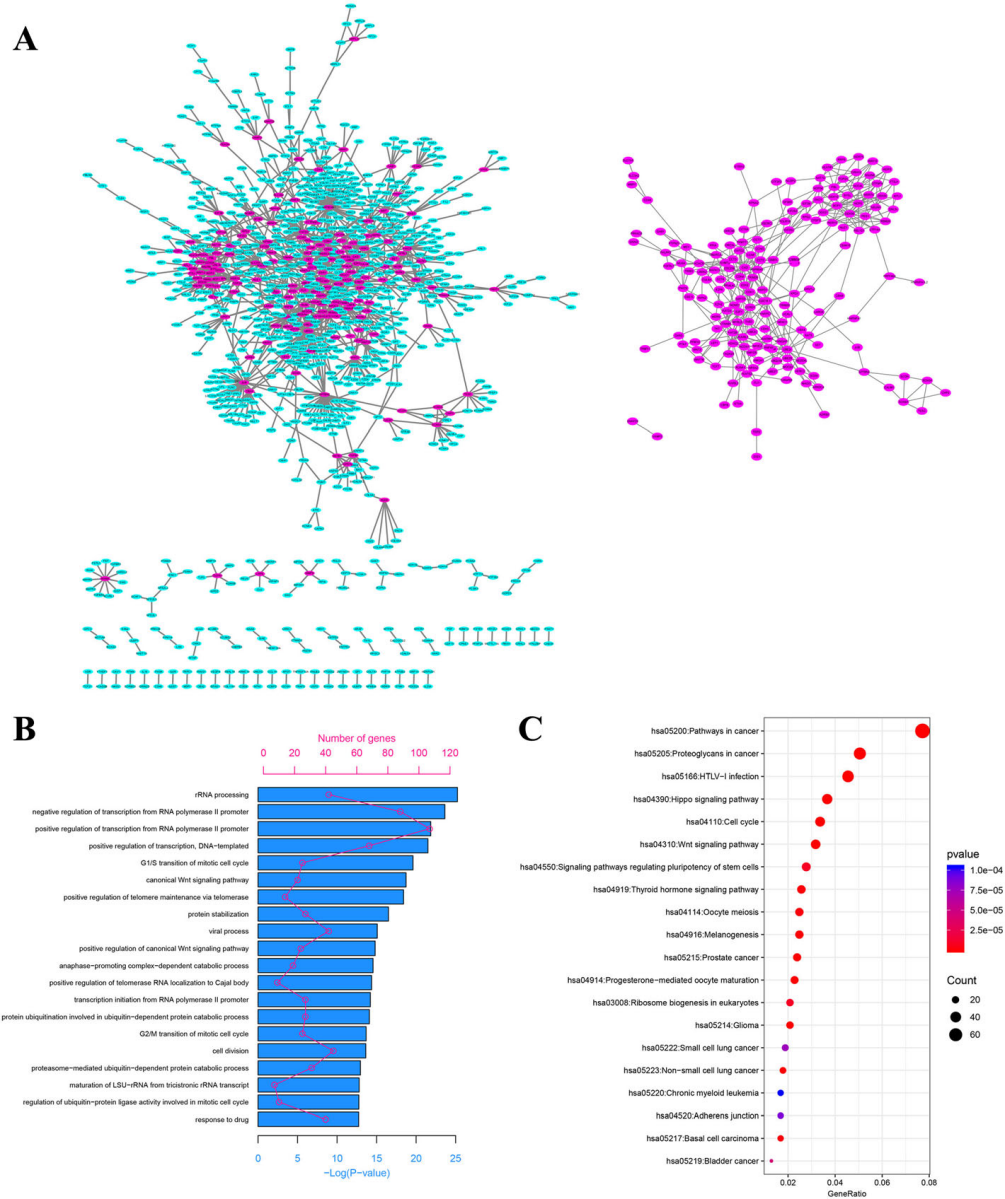
Functional network analysis of COAD. **a** Left panel: a functional network constructed for COAD with 1063 nodes (functional genes) and 1440 edges (functional interactions), which purple red nodes indicate the 185 core functional genes; right panel: the network formed by the core functional genes. **b** The top 20 significant enriched Gene Ontology for the 1063 functional genes. **c** The top 20 significant enriched KEGG pathways for the 1063 functional genes

Through the sample-specific network (SSN) method, SSNs were constructed for every tumor and normal sample with all 41 normal samples as reference samples (Fig. 1). Then, specific SSN for each tumor sample was constructed by deleting edges presented in SSNs of any normal samples. Finally, a functional network involving 1063 genes and 1440 edges was formed for COAD by collecting edges that appeared in more than 90% specific SSNs of tumor samples (Fig. 2a). The 1063 genes and 1440 edges in the functional network were regarded as functional genes and interactions, respectively. A histogram plot was drawn to show the distribution of node degrees in the functional network (Figure S2). Particularly, 185 genes with node degree over 3 were chosen as core functional genes (Fig. 2a).

### Enrichment analysis of functional genes

Functional genes were submitted for further enrichment analysis of GO and KEGG pathways with DAVID, respectively. The GO analysis of functional genes suggested that they were significantly enriched in rRNA processing, negative regulation of transcription from RNA polymerase II promoter, positive regulation of transcription from RNA polymerase II promoter, positive regulation of transcription, DNA-templated, G1/S transition of mitotic cell cycle, canonical Wnt signaling pathway and so on (Fig. 2b and Supplementary Table S2). In the KEGG pathway analysis, functional genes were significantly enriched in pathways in cancer, proteoglycans in cancer, cell cycle, Hippo signaling pathway, and Wnt signaling pathway (Fig. 2c and Supplementary Table S3). From both functional and pathway enrichment analysis, we can see that our identified functional genes are related to cancer.

GO and KEGG pathway analysis were also carried out for the 185 core functional genes. And it was shown for GO analysis that they were significantly enriched in rRNA processing, positive regulation of telomerase RNA localization to Cajal body, positive regulation of telomere maintenance via telomerase, positive regulation of protein localization to Cajal body, positive regulation of transcription from RNA polymerase II promoter and so on (Supplementary Table S4). KEGG pathway enrichment analysis suggested that the core functional genes were mainly related to ribosome biogenesis in eukaryotes, cell cycle, HTLV-I infection, Wnt signaling pathway, progesterone-mediated oocyte maturation and so on (Supplementary Table S5). The results suggested that the 185 core functional genes are likely to play important roles in COAD.

**Fig. 3:**
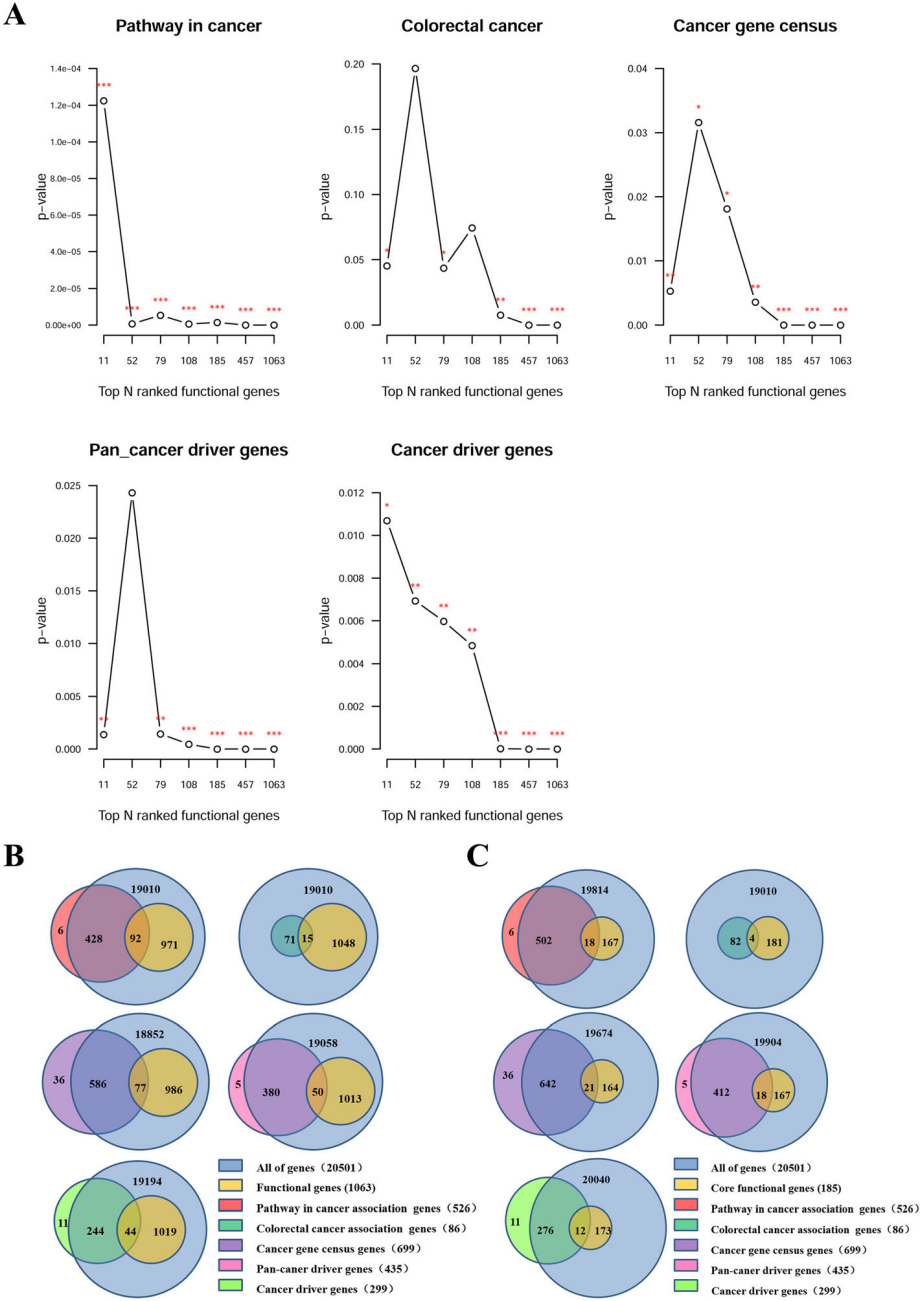
The relationship between functional genes and the five known cancer gene sets. **a** The *p*-value distribution for different top *N* ranked functional gene sets enrichment analysis with the five known cancer gene sets including the curated gene sets in pathway in cancer, colorectal cancer, cancer gene census, pan-caner driver genes, and cancer driver genes. Three stars (***) marks functional gene set whose *p*-value < 0.001, two stars (**) for functional gene set with *p*-value < 0.01, one star (*) for functional gene set with *p*-value < 0.05, and no stars for the one whose *p*-value > 0.05. **b** Venn plots for 1063 functional genes and the five known cancer gene sets. **c** Venn plots for the 185 core functional genes and the five known cancer gene sets

Furthermore, the *p*-values were calculated using the hypergeometric distribution (1) for different top *N* ranked functional gene sets enrichment analysis with the five known cancer gene sets including the curated gene sets in pathway in cancer, colorectal cancer, cancer gene census, pan-caner driver genes, and cancer driver genes. The different functional gene sets which were ranked by node degree included: top 11 functional genes with node degree over 19; top 52 functional genes with node degree over 9; top 79 functional genes with node degree over 7; top 109 functional genes with node degree over 5; top 185 functional genes with node degree over 3; top 457 functional genes with node degree over 1; and all 1063 functional genes. The results showed that almost every functional gene set was enriched in all the five known cancer gene sets except that the top 52 functional genes set was not enriched in colorectal cancer and pan-caner driver genes sets; top 108 functional gene set was not enriched in colorectal cancer set (Fig. 3a). Particularly, the 1063 functional genes were enriched in pathway in cancer (*p*-value = 0), colorectal cancer (*p*-value = 3.26 × 10^− 5^), cancer gene census (*p*-value = 3.17 × 10^− 10^), pan-caner driver genes (*p*-value = 1.16 × 10^− 7^), and cancer driver genes (*p*-value = 3.94 × 10^− 10^) (Fig. 3a). A Venn diagram was drawn to show the comparison of 1063 functional genes and the five known cancer gene sets (Fig. 3b). The results showed that 526 genes in pathway in cancer were obtained from KEGG, 92 of which appeared in functional genes; eighty-six genes in colorectal cancer were obtained from KEGG, 15 of which appeared in functional genes; six hundred and ninety-nine known cancer genes were obtained from the Cancer Gene Census database, 77 of which appeared in functional genes; four hundred and thirty-five pan-cancer driver genes were obtained from a pan-cancer study, 50 of which showed in functional genes; two hundred and ninety-nine driver genes were obtained from a comprehensive study of driver genes, 44 of which displayed in functional genes. And the 185 core functional genes were also enriched in pathway in cancer, colorectal cancer, cancer gene census, pan-caner driver genes, and cancer driver genes with *p*-value of 1.42 × 10^− 6^, 7.60 × 10^− 3^ 1.47 × 10^− 6^, 8.92 × 10^− 8^, 1.84 × 10^− 5^, respectively. Venn diagrams were drawn to show the comparison of 185 core functional genes and known cancer gene sets (Fig. 3c). From the results, we are confident to conclude that our identified functional genes are indeed correlated with cancers.

### Classification between tumor and normal samples by functional genes

**Fig. 4:**
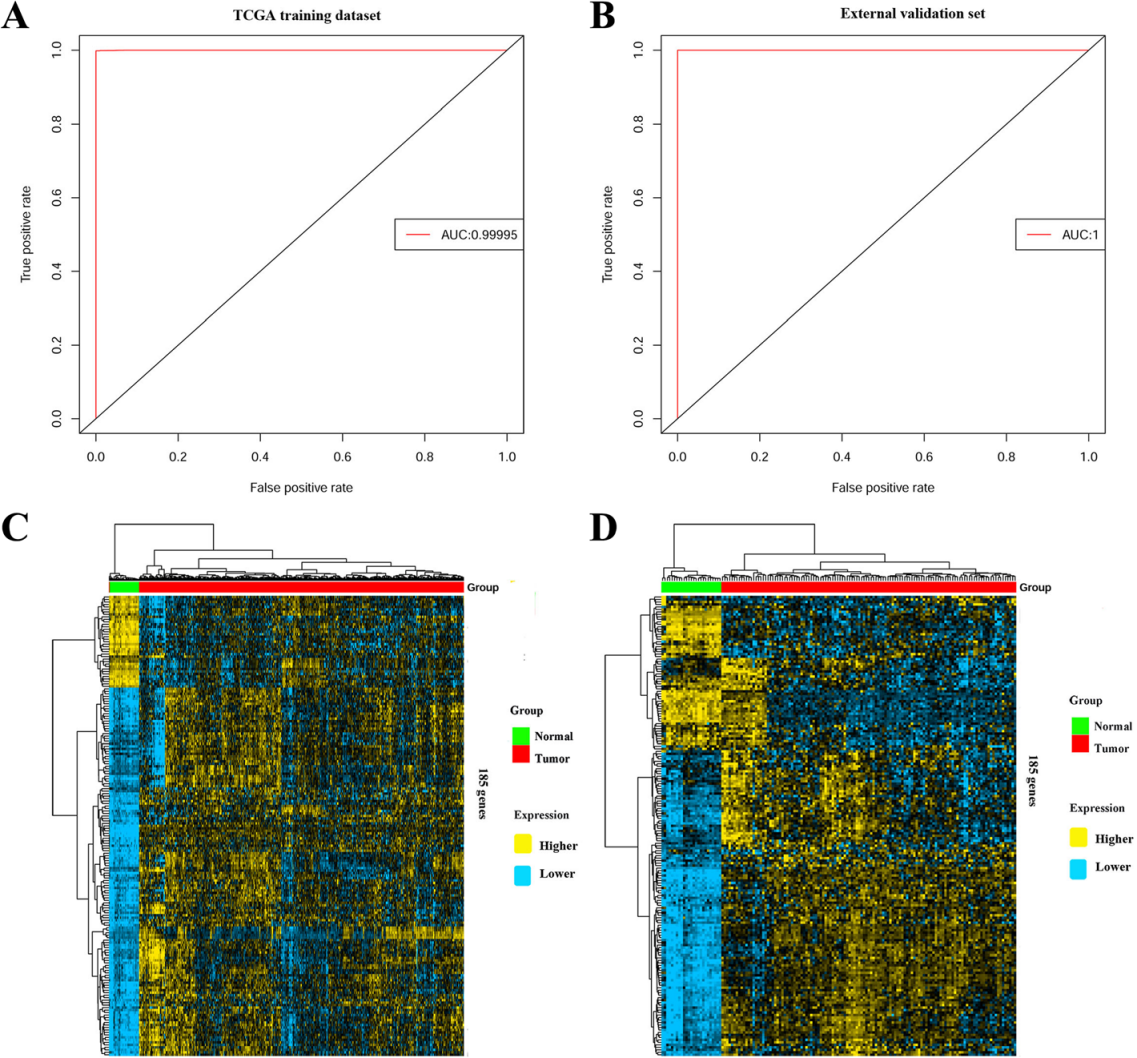
The results of classification between tumor and normal samples by 185 core functional genes. The figure shows ROC in the (**a**) training set (TCGA dataset) and (**b**) validation set (GSE21510 dataset). AUC: area under the curve. Heat maps show different expression patterns of 185 core functional genes between tumor and normal samples for (**c**) training set (TCGA dataset) and (**d**) validation set (GSE21510 dataset). The columns represent individual tissue samples covering the tumor (red) and normal samples (green). The rows represent individual genes. The heat map indicates up-regulation (yellow) and down-regulation (blue)

To investigate the ability of the 185 core functional genes to classify normal and tumor samples, we used an SVM model with 5-fold cross-validation to discriminate tumor samples from normal samples based on the expression profile of 185 core functional genes for both the training dataset (TCGA dataset) and independent validation dataset (GSE21510 dataset). ROC curves were drawn to show the prediction accuracy of the 185 core functional genes to discriminate tumor samples from normal samples (Fig. 4a-b). The results showed that the 185 core functional genes had a high area under the curve (AUC) for both the training dataset (AUC = 0.99994) and validation dataset (AUC = 1), indicating that the 185 core functional genes were potential biomarker candidates for COAD diagnosis.

Furthermore, hierarchical clustering was also performed using gene expression data of the 185 core functional genes for both the TCGA and validation datasets. The clustering results showed a high degree of separation of the tumor and normal samples by using the 185 core functional genes (Fig. 4c-d) in both the training and validation datasets. And the heatmap for 1063 functional genes in TCGA dataset was also drawn in Figure S3, which showed similar results with the 185 core functional genes. The classification results further confirmed that functional genes could be used as diagnostic biomarkers for COAD.

### SSN analysis uncovers major subtypes of COAD

Using gene expression of the core functional genes, consensus clustering method obtained 2 to 10 clusters. Then the k = 6 clustering solution was selected for further investigation. For the k = 6 clustering solution formed six different subtypes, referred to here as “c1” through “c6” (Table 1). The six subtypes of COAD included: c1 subtype with 38 cases (comprising 8.37% of tumor samples); c2 subtype with 138 cases (30.40%); c3 subtype with 99 cases (21.81%); c4 subtype with 85 cases (18.72%); c5 subtype with 38 cases (8.37%) and c6 subtype with 56 cases (12.33%) of COAD cases. The six subtypes could provide useful information about personalized medicine.

**Table 1:** Subtypes of COAD in TCGA Cohort

Subtype	Description	Therapeutic implications
c1	Cell cycle dysregulation; p53 signaling pathway; loss of *TCF7L2* and *RPL18P1*	Cell cycle; *p53; TCF7L2*; *RPL18P1*
c2	mTOR signing pathway and MAPK signaling pathway dysregulation	mTOR; MAPK
c3	Spliceosome; antigen processing and presentation; estrogen signaling pathway and mRNA surveillance pathway dysregulation; msi-h; high frequent *OBSCN*, *MYCBP2*, *RYR2* and *TTN* mutations	Spliceosome; antigen processing and presentation; estrogen; NMD; msi-h; *OBSCN*; *MYCBP2*; *RYR2*; *TTN*
c4	Viral infections dysregulation; spliceosome	Antiviral drugs; spliceosome
c5	Rig-I-like receptor signaling pathway; FoxO signaling pathway; autophagy; insulin signaling pathway; focal adhesion	Rig-I-like receptor signaling pathway; FoxO signaling pathway; autophagy; insulin signaling pathway; focal adhesion
c6	Glycosaminoglycan biosynthesis-heparan sulfate; tight junction; circadian rhythm; ECM-receptor interaction; dysregulation; loss of *ARHGEF28*, *BIN2P2* and *SLC25A5P9*	Glycosaminoglycans; protein Claudin-2; circadian rhythm; ECM-receptor interaction; *ARHGEF28*; *BIN2P2*; *SLC25A5P9*

**Fig. 5:**
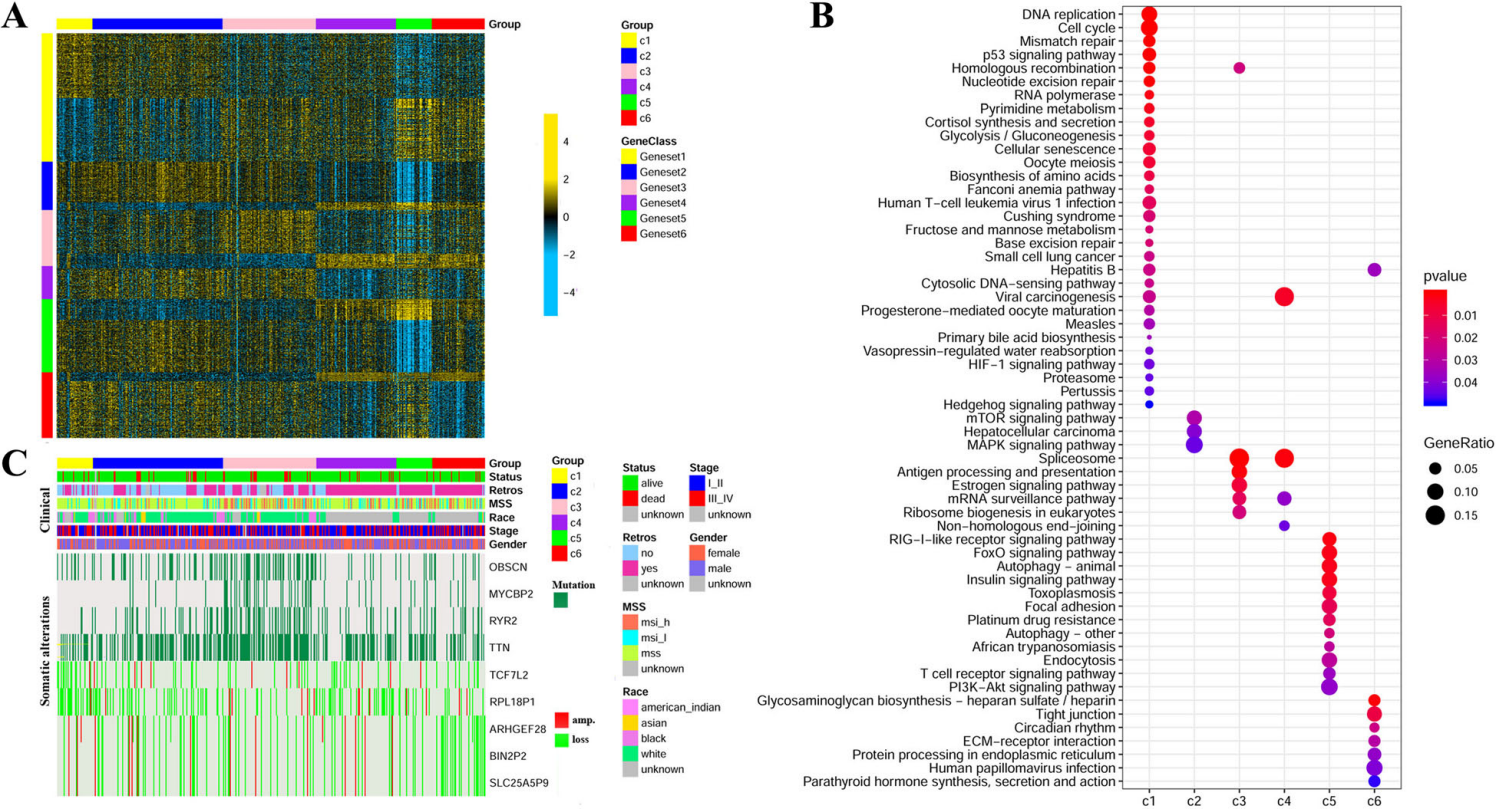
The six subtypes of COAD. **a** Differential gene expression patterns of a set of 1003 up- and down-regulated DEGs help to distinguish between the six subtypes. **b** Comparison of the enriched KEGG pathways of representative DEGs for different subtypes. **c** Different subtypes with different clinical and somatic alteration features. ‘Retros’ indicates ‘retrospective collection indicator’ (yes or no); microsatellite status (mss, MicroSatellite stability; msi-h, MicroSatellite Instability-High; msi-l, MicroSatellite Instability-Low); amp, amplification

The above subtypes were each characterized by different molecular patterns. For each of the six subtypes, the top 100 upregulated DEGs and top 100 downregulated DEGs were identified by comparing each subtype with the rest subtypes. For these detected top up- and down-regulated DEGs, the one which appeared in only one subtype was kept for each subtype. Finally, there were in all 1003 DEGs, including 161 up- and 157 down-regulated DEGs for subtype c1, 101 up- and 19 down-regulated DEGs for subtype c2, 108 up- and 31 down-regulated DEGs for subtype c3, 6 up- and 76 down-regulated DEGs for subtype c4, 51 up- and 130 down-regulated DEGs for subtype c5, 22 up- and 141 down-regulated DEGs for subtype c6. The heat map of the 1003 DEGs displayed different expression patterns for different subtypes (Fig. 5a). Finally, we used R packages clusterProfiler to compare these representative DEGs for each subtype by their enriched biological processes and KEGG pathways, with the cutoff of *p*-value< 0.05. As illustrated in Figure S4, representative DEGs for different subtypes related to different biological processes, such as representative DEGs for subtype c1 related to cell cycle, subtype c2 related to the regulation of GTPase activity, subtype c3 related to the regulation of cell division, subtype c4 related to the regulation of mRNA polyadenylation, subtype c5 related to autophagy and subtype c6 related to development. Furthermore, as shown in Fig. 5b, representative DEGs for different subtypes related to different KEGG pathways. Representative DEGs for subtype c1 were enriched in DNA replication, cell cycle, mismatch repair, and p53 signaling pathway and so on; representative DEGs for subtype c2 were enriched in mTOR signing pathway and MAPK signaling pathway; representative DEGs for subtype c3 were enriched in spliceosome, antigen processing and presentation, estrogen signaling pathway and mRNA surveillance pathway; representative DEGs for subtype c4 were enriched in viral carcinogenesis and spliceosome, and so on; representative DEGs for subtype c5 were enriched in Rig-I-like receptor signaling pathway, FoxO signaling pathway, autophagy-animal, insulin signaling pathway, toxoplasmosis, and focal adhesion, and so on; and representative DEGs for subtype c6 were enriched in glycosaminoglycan biosynthesis-heparan sulfate, tight junction, circadian rhythm, ECM-receptor interaction and so on. Therefore, the six subtypes showed different pathological mechanisms, which implied that they should be treated with different methods.

Most samples in subtype c4-c6 were retrospective samples, while many samples in subtype c2 were not retrospective samples (Fig. 5c). Most patients in subtype c1 showed mss (MicroSatellite stability), while many patients in subtype c3 showed msi-h (MicroSatellite Instability-High) feature (Fig. 5c). Many patients in both subtype c2 and c3 were white people (Fig. 5c). The six subtypes had a totally different meaning of tumor stages (Fig. 5c). The mutations of *OBSCN* (Obscurin), *MYCBP2* (*MYC* Binding Protein 2), *RYR2* (Ryanodine Receptor 2) and *TTN* (Titin) were most frequent in subtype c3 (Fig. 5c). Copy loss of *TCF7L2* (Transcription Factor 7 Like 2) and *RPL18P1* (Ribosomal Protein L18 Pseudogene 1) were frequent in subtype c1, while copy loss of *ARHGEF28* (Rho Guanine Nucleotide Exchange Factor 28), *BIN2P2* (Bridging Integrator 2 Pseudogene 2) and *SLC25A5P9* (Solute Carrier Family 25 Member 5 Pseudogene 9) were frequent in subtype c6 (Fig. 5c). It will provide recommendations for the treatment of the six subtypes of COAD with these identified subtype-specific clinical and somatic alteration features.

**Fig. 6:**
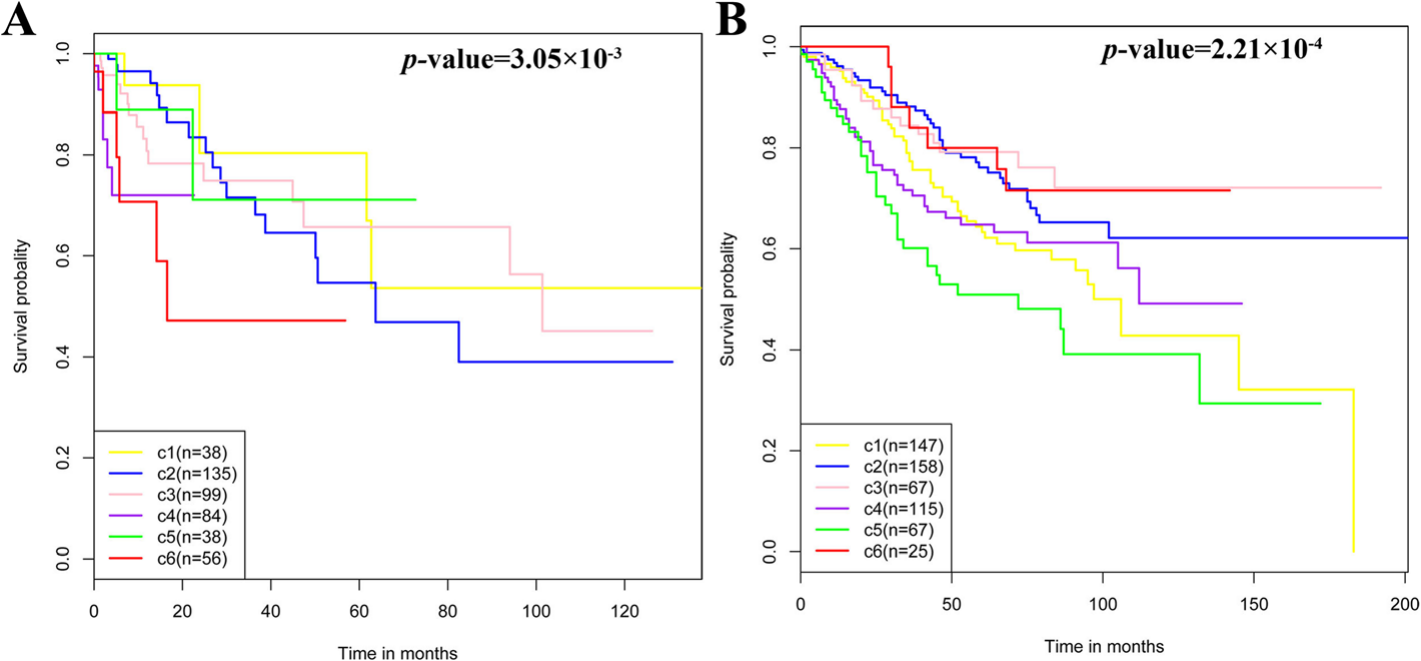
Survival analysis results for six subtypes of COAD. **a** Survival curves for six subtypes of COAD in TCGA dataset. **b** Survival curves for six subtypes of COAD in the external dataset (GSE39582)

Survival analysis was performed on 450 tumor samples with clinical data (Fig. 6a). Significant survival differences between the six subtypes were observed (Fig. 6a, *p*-value = 3.05 × 10^− 3^, log-rank), suggesting that the classification showed biological significance. To further validate the results, survival analysis was further performed in an external dataset (GSE39582), which also showed survival differences between six subtypes (Fig. 6b, *p*-value = 2.21 × 10^− 4^, log-rank). Both results suggested that patients in different subtypes had different survival rates, which may help doctors develop rational treatments for patients based on the subtypes to which they belong.

### Specific functional genes and edges associated with survival

**Fig. 7:**
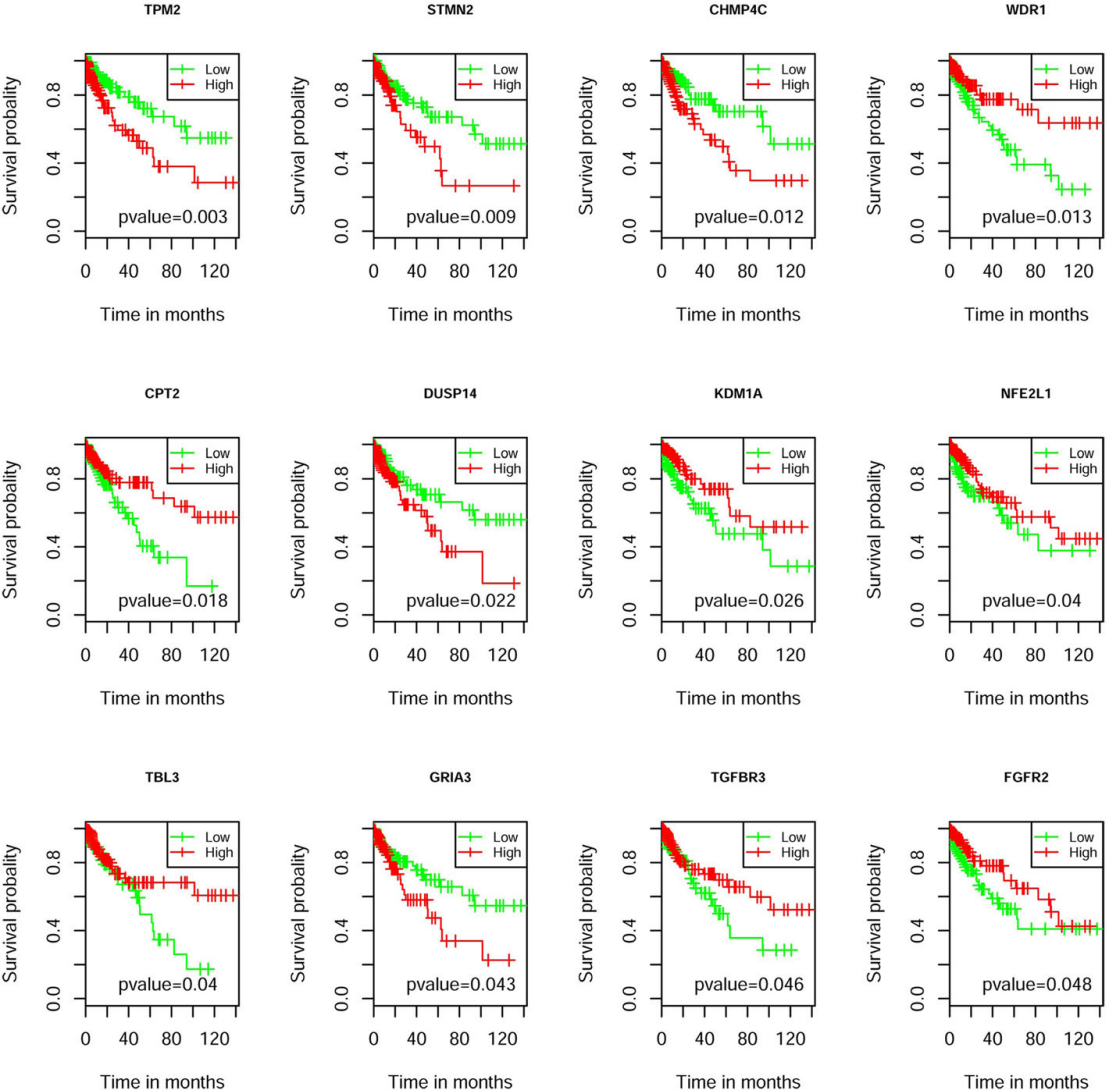
The survival plots for 12 functional genes

To further investigate the potential of functional genes and interactions as prognosis biomarkers for COAD. All 1063 functional genes and 1440 functional interactions were analyzed for their prognostic significance of overall survival. For each functional gene, all COAD patients were classified into the low- or high- expression group, according to the median expression level. For each functional edge, patients were also classified into the low- or high- *ΔPCC*s group based on the median *ΔPCC*s level for the functional edge. Functional genes and interactions with *p*-value less than 0.05 in the log-rank test for both univariate analysis and multivariate analysis were selected as prognosis biomarkers for COAD. Survival analysis suggested that 12 functional genes and 13 functional interactions were associated with the overall survival of patients of COAD (Fig. 7 and Figure S5, Tables S6-S7), which demonstrates that they could be prognosis biomarkers for COAD.

## Discussion

Our study constructed a functional network of COAD based on sample-specific network theory. The results showed that the nodes in the functional network which we denoted as functional genes had the potential roles in discriminate tumor samples from normal samples, COAD subtyping and prognosis. And the edges in the functional network which we called functional interactions could be prognosis biomarkers for COAD.

The enrichment analysis for the 1063 functional genes revealed some key biological processes and pathways which could play roles in pathogenesis and progression of cancer (Figure S6). Specifically, among the top 5 most enriched GO terms (Figure S6A), rRNA processing as the most enriched one involved in 42 functional genes that were upregulated in COAD compared with normal samples. And upregulation of rRNA processing genes was reported to be connected with CRC, which can overproduce the matured ribosomal structures in CRC [26]. The next three most enriched GO terms included “negative regulation of transcription from RNA polymerase II promoter”, “positive regulation of transcription from RNA polymerase II promoter” and “positive regulation of transcription, DNA-templated”, play important roles in regulating the process of transcription. The term “G1/S transition of mitotic cell cycle” contained 25 functional genes. And 23 of the 25 functional genes were significantly up-regulated in COAD, such as *CDK1*, *CDK2*, *CDK4*, *CDK6*, and *CDK7*, which can cause uncontrolled proliferation and may serve as promising targets in cancer therapy [27]. The top 5 most enriched KEGG pathways were shown in Figure S6B. Among them, pathways in cancer, proteoglycans in cancer, and cell cycle are correlated with cancers. The deregulation of Hippo signaling pathway was found in CRC and the interaction between Hippo and Wnt signaling play crucial roles in CRC development [28]. Wnt signaling pathway plays important roles in CRC and could be a potential target of revolutionary therapeutic treatments for CRC [29]. Therefore, the references confirmed the importance of the 5 most enriched GO and KEGG pathways of the 1063 functional genes.

Furthermore, our results demonstrated that the 1063 functional genes were enriched in the five known cancer gene sets including the curated gene sets in pathway in cancer, colorectal cancer, cancer gene census, pan-caner driver genes, and cancer driver genes, which also implied the important roles of the 1063 functional genes in COAD.

Literature searches were conducted to further investigate the functions of the top 20 functional genes with the highest node degree, which found that 11 genes were related to CRC (Table S8). In addition, four genes (*CCND1*, *WNT2*, *MET*, and *HDAC2*) of the 11 genes were contained by the five known cancer gene sets (Table S8). Specifically, CyclinD1 (*CCND1*) polymorphisms were associated with CRC [30]; *WNT2*, a member of the WNT gene family, is involving in a signaling pathway which can promote colorectal cancer progression [31]; *MET* (MET Proto-Oncogene) may act as prognosis biomarkers for CRC [32]; *HDAC2* (Histone Deacetylase 2) was found to be a potential target in CRC [33]. Besides, literature searches found that our method could also identify new biomarkers not contained by the five know cancer gene sets. For example, *UBE2I*, the small ubiquitin-like modifier (SUMO) E2 ligase, was reported as a critical factor in sustaining the transformation growth of KRAS mutant colorectal cancer cells, which suggested that *UBE2I* could be a drug target for the treatment of KRAS mutant colorectal cancers; LIM Protein *JUB* was reported as a novel target for the therapy of metastatic CRC since it is a tumor-promoting gene which can promote Epithelial-mesenchymal transition (EMT) [34]; ubiquitin-conjugating enzyme E2S (*UBE2S*) was reported as a potential target for CRC therapy since it plays an important role in determining malignancy properties of human CRC cells [35]; the atypical cyclin *CNTD2* which can promote colon cancer cell proliferation and migration, was reported as a new prognostic factor and drug target for CRC [36]; it has been found that *TRIB3* (Tribbles Pseudokinase 3) may act as prognosis biomarker for CRC [32]; *BOP1* (BOP1 Ribosomal Biogenesis Factor) is responsible for the colorectal tumorigenesis [37]; *GTPBP4* (GTP Binding Protein 4) is involved in the metastasis of CRC [38]. The results proved that our identified functional genes not only contained the known cancer genes but also included the important genes related to CRC.

Gene expression data can be used to realize the classification between tumor and normal samples, which may suggest targeted therapy options. We carried out a classification of COAD tumor from normal samples using the gene expression data of functional genes. The high prediction accuracy reached by the 185 core functional genes to discriminate tumor from normal samples in both TCGA dataset and independent validation dataset, and it suggested that functional genes were potential diagnostic biomarkers for COAD.

Six subtypes of COAD were detected by using consensus clustering method based on the expression profile of 185 core functional genes, including subtype c1 (*n* = 38), subtype c2 (*n* = 138), subtype c3 (*n* = 99), subtype c4 (*n* = 85), subtype c5 (*n* = 38) and subtype c6 (*n* = 56). For subtype c1, 318 DEGs (161 up-regulated and 157 down-regulated) were associated with subtype c1, enriched in many important pathways such as DNA replication, cell cycle, mismatch repair, and p53 signaling pathway, and so on, which suggested that subtype c1 had abnormal cell cycle process and p53 signaling pathway dysregulation. Besides, subtypes c1 had the characteristic of high frequent copy loss of *TCF7L2* which can promote migration and invasion of human colorectal cancer cells reported by the latest study [39]. High frequent copy loss of *RPL18P1* was also found in subtype c1, which could also play important roles in subtype c1. Consequently, our founding suggested that we can focus on cell cycle, *p53*, *TCF7L2*, *RPL18P1* when finding therapeutic drugs for subtype c1. For subtype c2, 120 DEGs (101 up-regulated and 19 down-regulated) were detected as representative genes, which were enriched mTOR signing pathway and MAPK signaling pathway. It is well known that both mTOR signing pathway and MAPK signaling pathway are two of the most implicated cellular pathways in cancers. In addition, Todd M.P. et al. demonstrated that the combination of a PI3K/mTOR and a MAPK inhibitor can enhance anti-proliferative effects against CRC cell lines [40] and Wang H. et al. reported that targeting mTOR suppresses colon cancer growth [41], which suggested that mTOR and MAPK could be therapeutic targets for subtype c2. For subtype c3, 139 DEGs (108 up-regulated and 31 down-regulated) were identified as representative genes, which were enriched in spliceosome, antigen processing and presentation, estrogen signaling pathway and mRNA surveillance pathway. The spliceosome pathway was reported as a target for anticancer treatment [42] and displayed phase-shifted circadian expression in CRC [43]. Downregulated antigen processing and presentation were reported in CRC [44]. Estrogen signaling pathway was reported as a target for colorectal cancer [45]. mRNA surveillance pathway is to detect and degrade abnormal mRNAs. Nonsense-mediated mRNA decay (NMD) as one of mRNA surveillance pathway has been reported as a target for colorectal cancers with microsatellite instability [46]. Besides, many patients in subtype c3 showed msi-h feature and had high frequent *OBSCN*, *MYCBP2*, *RYR2* and *TTN* mutations. It was reported that msi-h could be a potential prognostic and therapeutic factor for COAD [47], which suggested that msi-h could play important roles for the patients in subtype c3 with msi-h. *OBSCN*, *RYR2* and *TTN* mutations which have been reported as drivers [48] could be biomarkers for subtype c3. And more, *MYCBP2* was reported as a potential therapeutic target for CRC [49], which could offer treatment suggestions for subtype c3. Therefore, for patients in subtype c3, spliceosome, antigen processing and presentation, estrogen signaling pathway, NMD, msi-h, *OBSCN*, *MYCBP2*, *RYR2*, and *TTN* could be the potential therapeutic targets. For subtype c4, 82 DEGs (6 up-regulated and 76 down-regulated) were found as representative genes that were enriched in viral carcinogenesis and spliceosome, and so on. Viral carcinogenesis is a factor to induce DNA damage and virus integration [50] and may be involved in the etiology of CRC [51]. Hence, viral carcinogenesis and spliceosome could be the potential targets for subtype c4. For subtype c5, 181 DEGs (51 up-regulated and 130 down-regulated) were detected and were enriched in Rig-I-like receptor signaling pathway, FoxO signaling pathway, autophagy-animal, insulin signaling pathway, toxoplasmosis, and focal adhesion, and so on. Among the enriched pathways, RIG-I-like receptor signaling plays important roles in colon cancer [52]; FoxO signaling pathway has been reported as therapeutic targets in cancer [53]; autophagy was reported as a promising target for CRC [54]; insulin signaling pathway could be a potential CRC therapy [55]. In consequence, these pathways could be the targets for subtype c5. For subtype c6, 163 DEGs (22 up-regulated and 141 down-regulated) were identified as representative genes and were enriched in glycosaminoglycan biosynthesis-heparan sulfate, tight junction, circadian rhythm, ECM-receptor interaction and so on. Glycosaminoglycans have therapeutic value in cancer [56]; tight junction whose protein claudin-2 has been reported as a potential target for CRC therapy [57]; circadian rhythm plays roles in the pathogenesis of CRC [58]; ECM-receptor interaction may play a critical role in CRC metastasis [59]. In addition, copy loss of *ARHGEF28*, *BIN2P2*, and *SLC25A5P9* were frequent in subtype c6, which suggested that they may be the potential biomarkers. Therefore, glycosaminoglycans, protein Claudin-2, circadian rhythm, ECM-receptor interaction, *ARHGEF28*, *BIN2P2*, and *SLC25A5P9* could provide information for the treatment of subtype c6. Taken together, these findings suggested that distinct subtypes of COAD could be treated with specific targeted therapies (Table 1).

Among the 12 functional genes which were associated with the prognosis of COAD, high expression of *TPM2*, *STMN2*, *CHMP4C*, *DUSP14*, and *GRIA3* had poorer survival rates, while low expression of *WDR1*, *CPT2*, *KDM1A*, *NFE2L1*, *TBL3*, *TGFBR3*, and *FGFR2* had worse survival rates. Some of the 12 functional genes have been connected with COAD or other diseases according to the existing research. For example, *TPM2* was reported to be in implicated in CRC [60]; *STMN2* might be involved in beta-catenin/TCF-mediated carcinogenesis in human hepatoma cells [61]; *CHMP4C* was identified as a novel molecular target gene for ovarian cancer [62]; *GRIA3* may act as a mediator of tumor progression in pancreatic cancer [63]; *WDR1* was reported as a therapeutic target in lung cancer [64]; *CPT2* was identified as a potential diagnostic biomarker of colon cancer [65]; Somatic deletion of *KDM1A* plays role in advanced colorectal cancer stages [66]; *NFE2L1*, also called Nrf1, was found to be associated to high-risk diffuse large B cell lymphoma [67]; Gatza et al. reported that *TGFBR3* promotes colon cancer progression [68]; *FGFR2* was shown to promote gastric cancer progression [69]. Therefore, the 12 functional genes probably play important roles in COAD and could be the potential prognosis biomarkers for COAD. There was no obvious correlation between the expression of 12 genes (Figure S7). To find the best combination of them, we performed LASSO Cox regression on the 12 functional genes to select the most informative gene set for prognosis (Figure S8). Eventually, seven functional genes (*CHMP4C*, *WDR1*, *CPT2*, *DUSP14*, *NFE2L1*, *TBL3*, and *TGFBR3*) were selected as the most informative gene set for prognosis. The *p*-value was 3.00 × 10^− 4^ for the best model with the seven genes in cox analysis which was better than only use one gene model.

The 13 functional interactions which could be potential prognosis biomarkers provides a new suggestion for cancer prognosis. And LASSO Cox regression was also performed for the 13 functional edges, resulting in seven functional interactions (*ESR1*_*E2F1*, *ARRDC4*_*HECTD3*, *SPTBN2*_*SPTAN1*, *SOX9*_*UBE2I*, *CBX8*_*HOXA9*, *PPM1G*_*STMN2*, *E2F1*_*KDM1A*) were selected as the most informative edge set for prognosis with *p*-value = 4.00 × 10^− 6^. It is worth pointing out that Narayanan S.P. et al. found that *KDM1A* plays a role in cell proliferation through regulating the E2F1 signaling pathway in oral cancer [70] and *CBX8* interaction with *HOXA9* was found to play an important role in MLL-AF9-Induced Leukemogenesis [71], which suggested that they may also play important roles in COAD.

The main limitations of the study are: the biomarkers and subtypes detected in this study need to be proved with more external datasets and biological experiments; the roles of the functional network as a whole need to be further explained.

## Conclusions

In this study, a functional network with 1063 nodes and 1440 edges was constructed for COAD by a sample-specific network (SSN) method. The roles of the nodes and edges of the functional network which were defined as functional genes and interactions were further explored. The results showed that the functional genes could be used as diagnostic biomarkers. The consensus clustering method was used to classify COAD into six subtypes (c1-c6). The representative genes of each subtype could be used as potentially targetable markers for each subtype. Different subtypes were characterized by different molecular patterns including clinical and mutation features which provide a therapeutic suggestion for each subtype. The last but not least, 12 functional genes and 13 functional interactions that were associated with the overall survival of COAD could serve as prognosis biomarkers. Therefore, our study could help to realize the personalized treatment of COAD.

## Supplementary information

**Additional file 1 Figure S1.** The summary of the mutation drawn by maftools, with six plots that represent descriptive features of the mutations and their annotations. **Figure S2.** A histogram plot showing the distribution of node degrees in the functional network. **Figure S3.** The classification of cancer samples (454 samples, the red bar) and normal samples (41 samples, the green bar) by hierarchical clustering the expression of 1063 functional genes. The columns represent individual tissue samples covering tumor and normal samples and the rows represent individual genes. The heat map indicates up-regulation (burgundy) and down-regulation (sky blue). **Figure S4.** Comparison of GO enrichment of representative DEGs for different subtypes. **Figure S5.** The survival plots for 13 functional interactions. **Figure S6.** Enrichment analysis for 1063 functional genes. (A) The top 5 significant enriched GO for 1063 functional genes. (B) The top 5 significant enriched KEGG pathways for 1063 functional genes. **Figure S7.** The pair-wise correlations between the 12 functional genes. **Figure S8.** LASSO regression results. The plot of partial likelihood deviance for the 12 functional genes in TCGA cohort.

**Additional file 2 Table S1.** Summary of Patient Cohort Information. **Table S2.** Top 20 Most Enriched Functions of the 1063 Functional Genes. **Table S3.** Top 20 Most Enriched KEGG Pathways of the 1063 Functional Genes. **Table S4.** Top 20 Most Enriched Functions of the 185 Core Functional Genes**. Table S5.** Top 20 Enriched KEGG Pathways of the 185 Core Functional Genes**. Table S6.** Twelve Functional Genes Associated with the Prognosis of COAD. **Table S7.** Thirteen Functional Interactions Associated with the Prognosis of COAD. **Table S8.** Eleven Genes Related to Colorectal Cancer.

## Data Availability

The datasets used to perform the analysis are publicly available at http://cancergenome.nih.gov/, https://gdc.cancer.gov/, https://www.ncbi.nlm.nih.gov/gds/, and http://string-db.org/.

## Abbreviations

COAD: Colon adenocarcinoma
SSN: Sample-specific network
CRC: Colorectal cancer
GO: Gene ontology
KEGG: Kyoto encyclopedia of genes and genomes
MAF: Mutation annotation format
TCGA: The cancer genome atlas
GEO: Gene expression omnibus
PCC: Pearson correlation coefficient
SVM: Support vector machine
ROC: Receiver operating characteristic
DEGs: Differentially expressed genes
AUC: Area under the curve
mss: MicroSatellite stability
msi-h: MicroSatellite Instability-High
EMT: Epithelial-mesenchymal transition
NMD: Nonsense-mediated mRNA decay
