# Supplementary material for: Network-based identification of biomarkers for colon adenocarcinoma
Source: BMC Cancer. 2020 Jul 17;20:668. doi: 10.1186/s12885-020-07157-w (PMC7367377; doi:10.1186/s12885-020-07157-w)
Supplement: Supplementary file 1 — Additional file 1 Figure S1. The summary of the mutation drawn by maftools, with six plots that represent descriptive features of the mutations and their annotations. Figure S2. A histogram plot showing the distribution of node degrees in the functional network. Figure S3. The classification of cancer samples (454 samples, the red bar) and normal samples (41 samples, the green bar) by hierarchical clustering the expression of 1063 functional genes. The columns represent individual tissue samples covering tumor and normal samples and the rows represent individual genes. The heat map indicates up-regulation (burgundy) and down-regulation (sky blue). Figure S4. Comparison of GO enrichment of representative DEGs for different subtypes. Figure S5. The survival plots for 13 functional interactions. Figure S6. Enrichment analysis for 1063 functional genes. (A) The top 5 significant enriched GO for 1063 functional genes. (B) The top 5 significant enriched KEGG pathways for 1063 functional genes. Figure S7. The pair-wise correlations between the 12 functional genes. Figure S8. LASSO regression results. The plot of partial likelihood deviance for the 12 functional genes in TCGA cohort. [file 12885_2020_7157_MOESM1_ESM.docx]

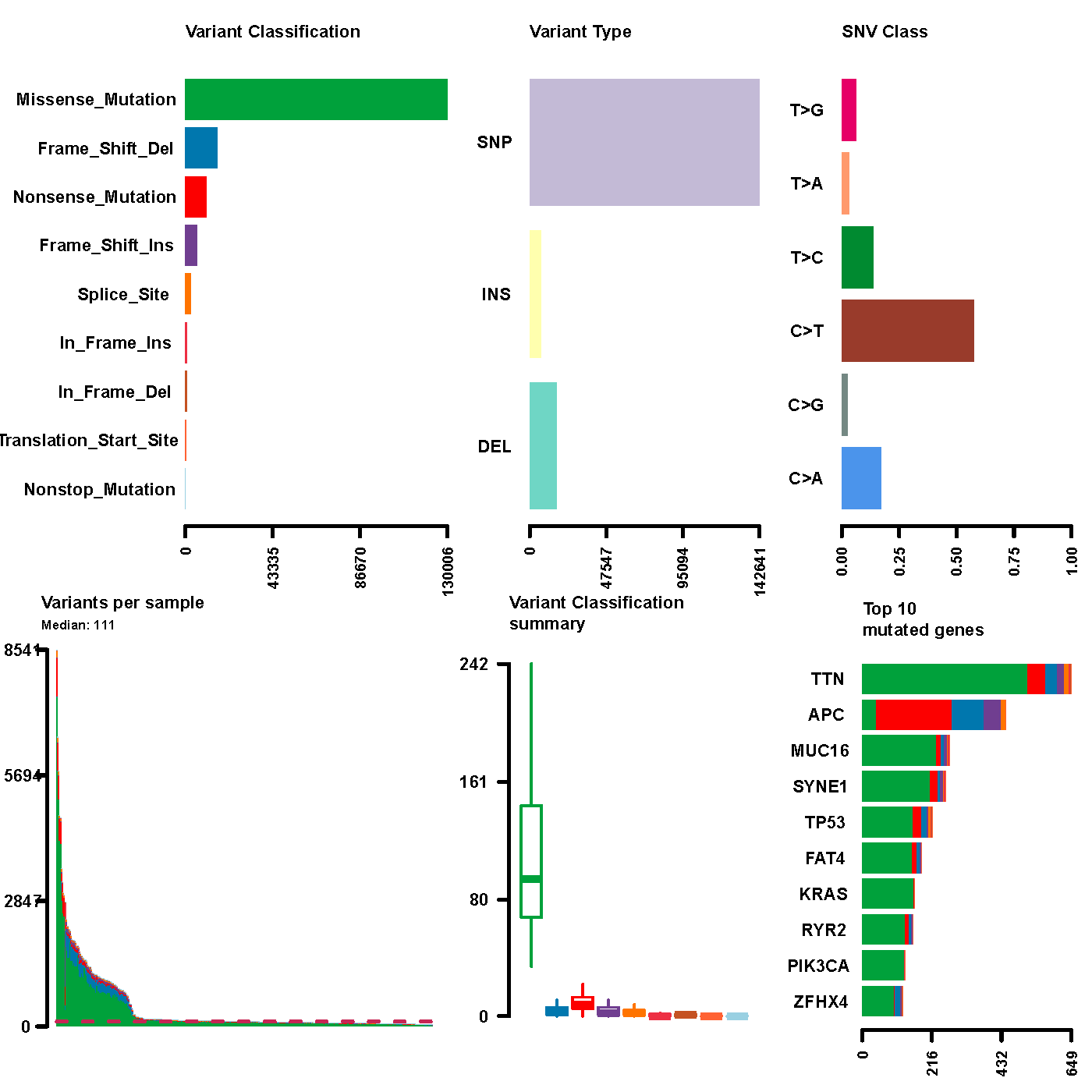


**Figure S1.** The summary of the mutation drawn by maftools, with six plots that represent descriptive features of the mutations and their annotations.


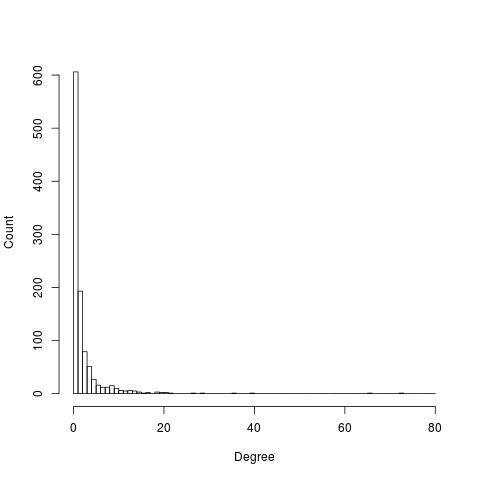


**Figure S2.** A histogram plot showing the distribution of node degrees in the functional network.


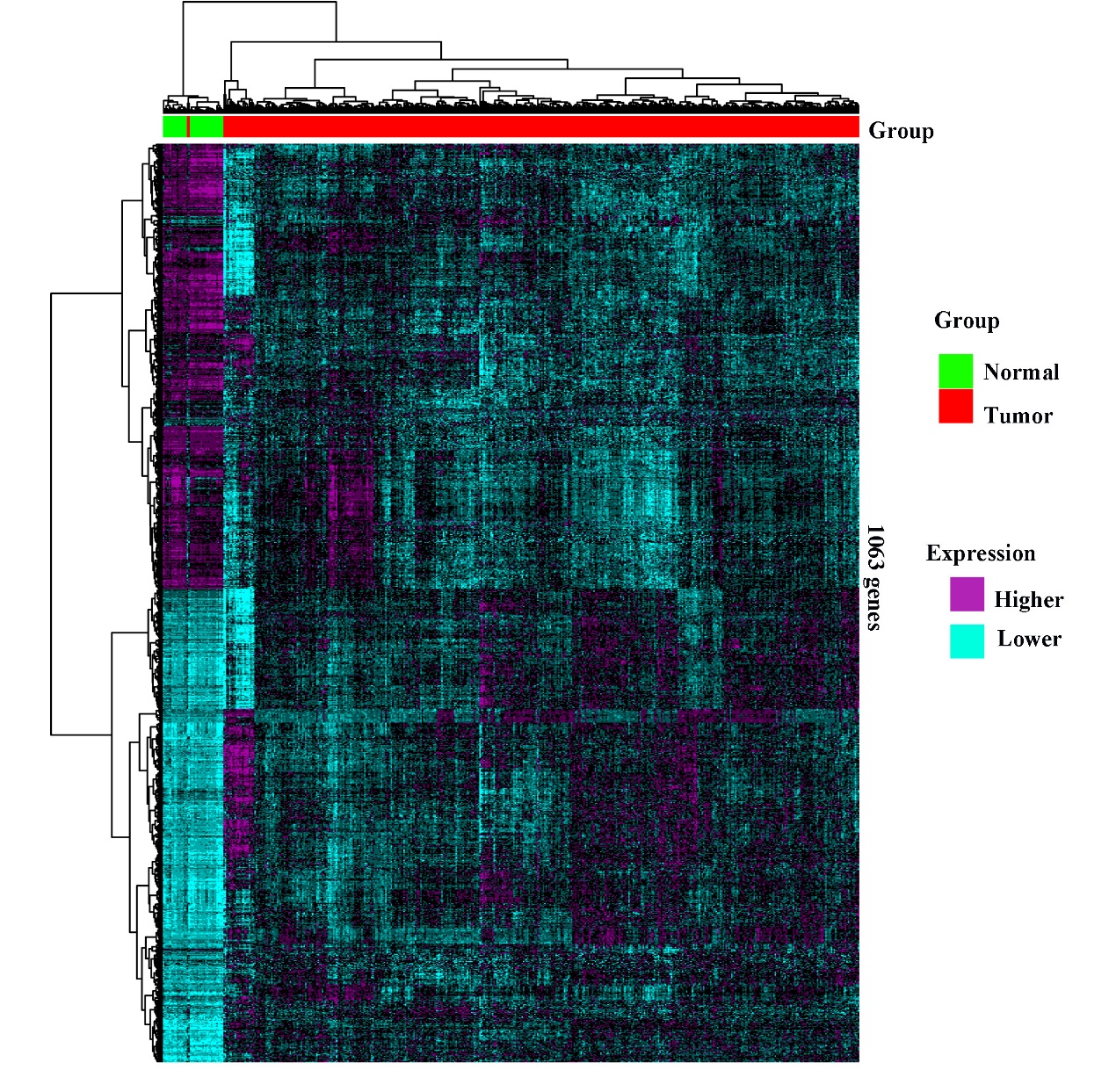


**Figure S3.** The classification of cancer samples (454 samples, the red bar) and normal samples (41 samples, the green bar) by hierarchical clustering the expression of 1063 functional genes. The columns represent individual tissue samples covering tumor and normal samples and the rows represent individual genes. The heat map indicates up-regulation (burgundy) and down-regulation (sky blue).


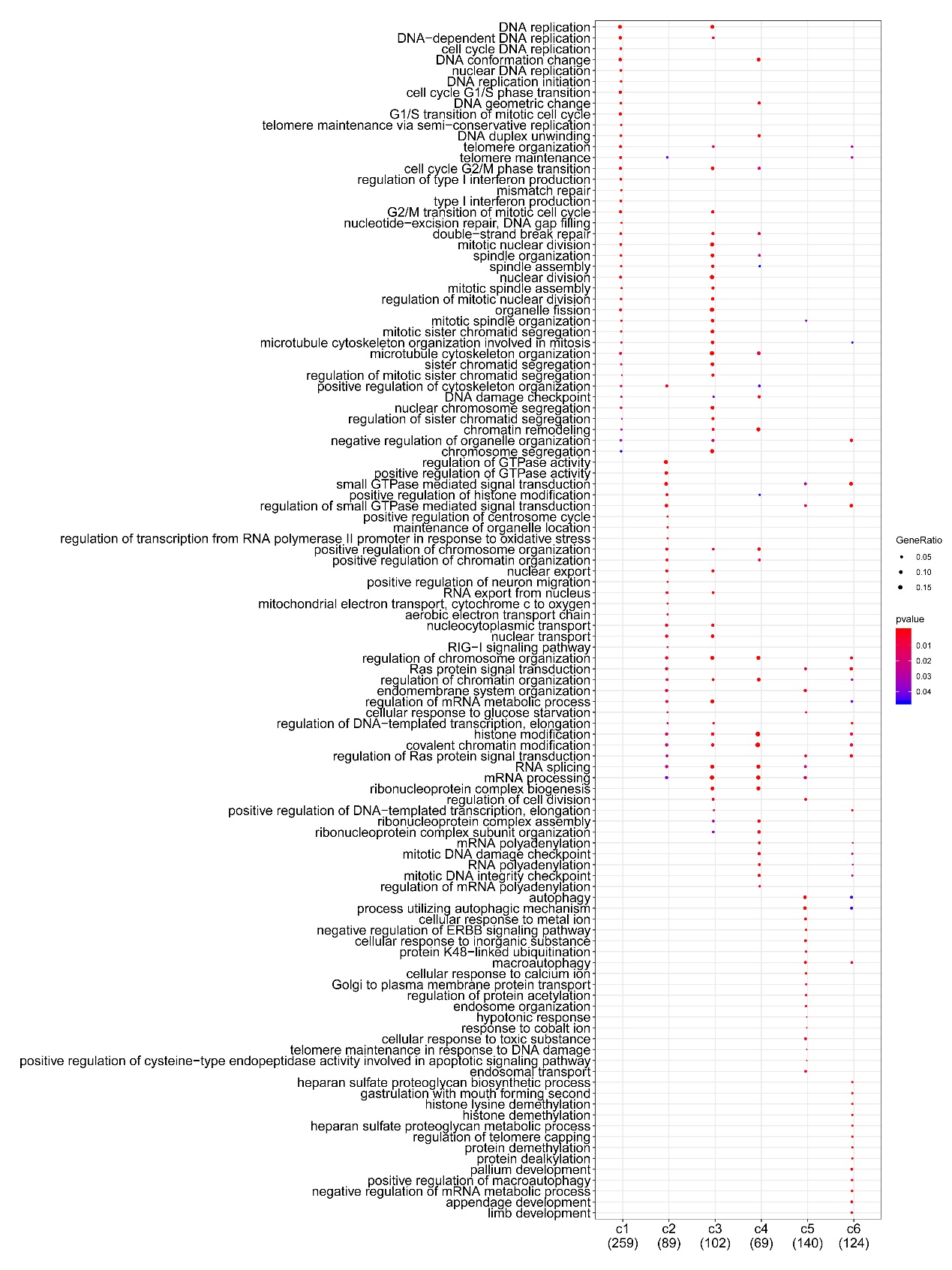


**Figure S4.** Comparison of GO enrichment of representative DEGs for different subtypes.


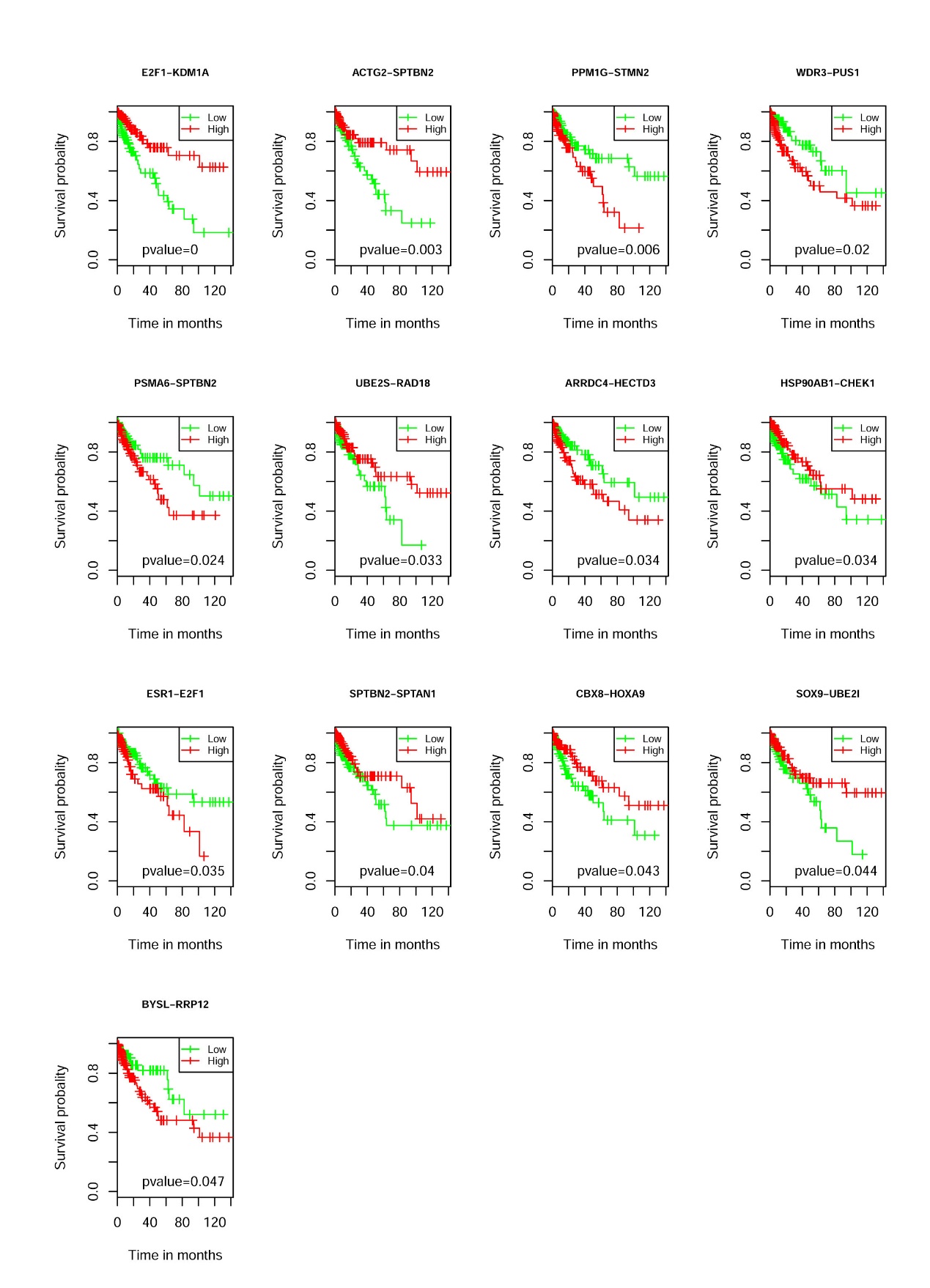


**Figure S5.** The survival plots for 13 functional interactions.


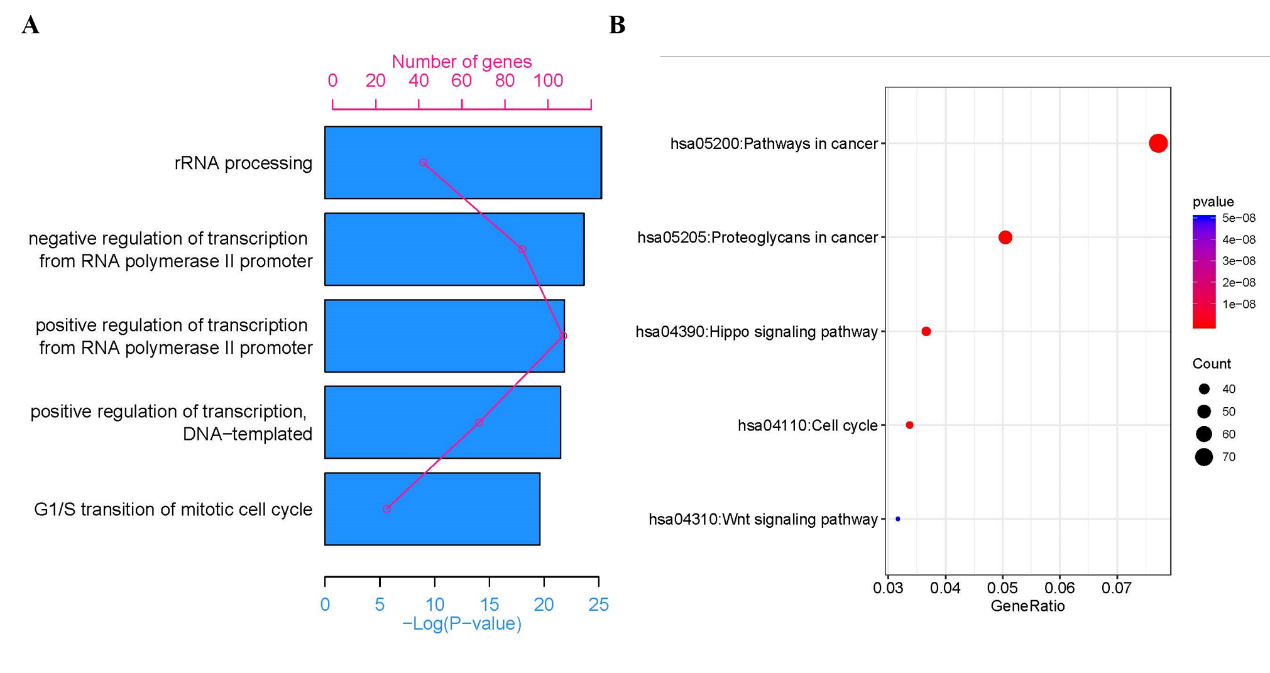


**Figure S6.** Enrichment analysis for 1063 functional genes. (A) The top 5 significant enriched GO for 1063 functional genes. (B) The top 5 significant enriched KEGG pathways for 1063 functional genes.

**
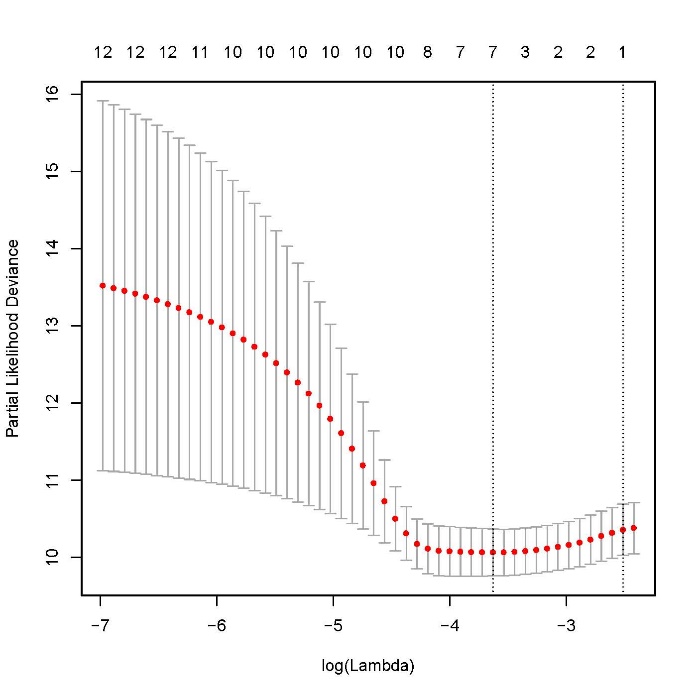

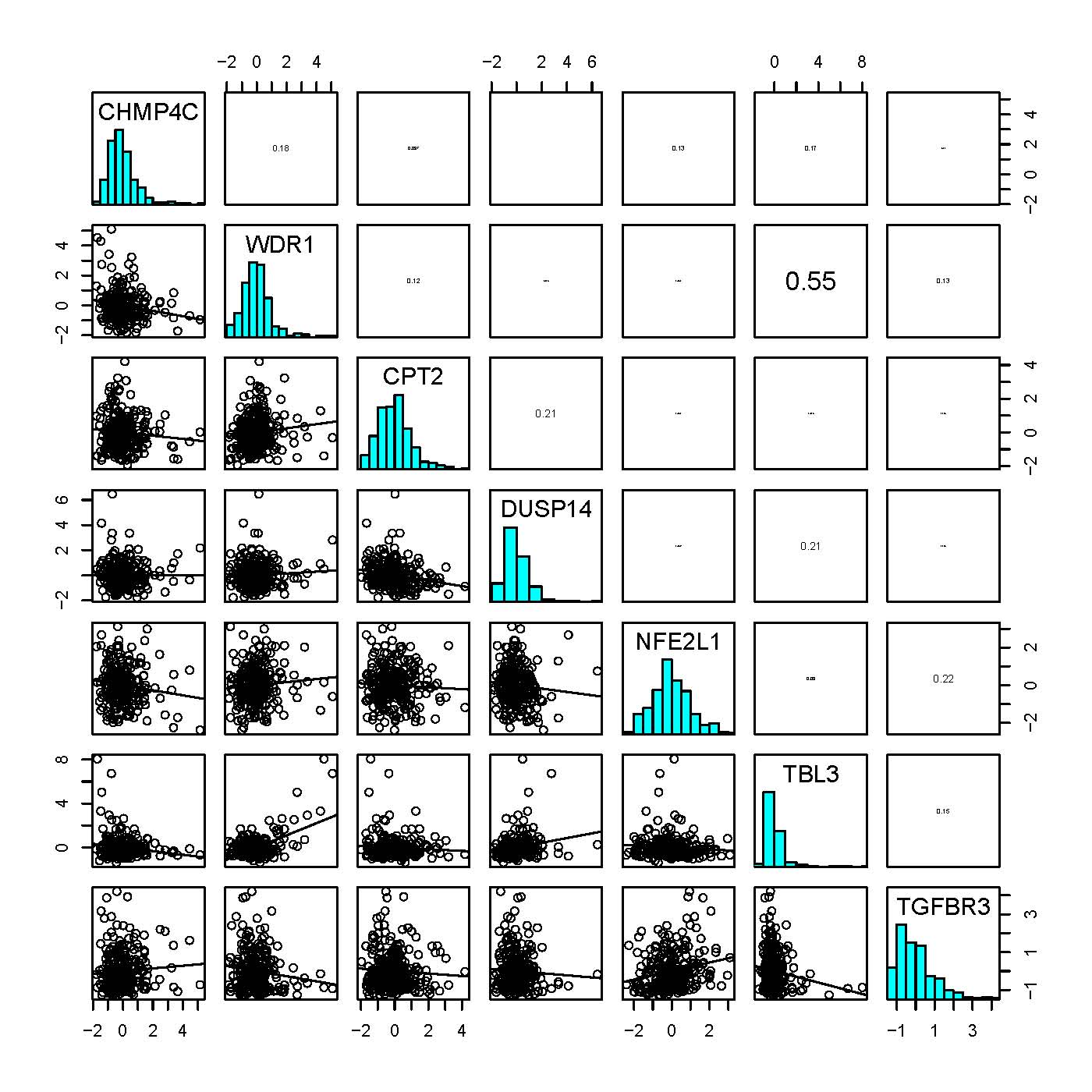
F****igure S7.** The pair-wise correlations between the 12 functional genes.

**Figure S8.** LASSO regression results. The plot of partial likelihood deviance for the 12 functional genes in TCGA cohort.
