# Supplementary material for: Network-based identification of biomarkers for colon adenocarcinoma
Source: BMC Cancer. 2020 Jul 17;20:668. doi: 10.1186/s12885-020-07157-w (PMC7367377; doi:10.1186/s12885-020-07157-w)
Supplement: Supplementary file 2 — Additional file 2 Table S1. Summary of Patient Cohort Information. Table S2. Top 20 Most Enriched Functions of the 1063 Functional Genes. Table S3. Top 20 Most Enriched KEGG Pathways of the 1063 Functional Genes. Table S4. Top 20 Most Enriched Functions of the 185 Core Functional Genes. Table S5. Top 20 Enriched KEGG Pathways of the 185 Core Functional Genes. Table S6. Twelve Functional Genes Associated with the Prognosis of COAD. Table S7. Thirteen Functional Interactions Associated with the Prognosis of COAD. Table S8. Eleven Genes Related to Colorectal Cancer. [file 12885_2020_7157_MOESM2_ESM.docx]

## **Table S1.** Summary of Patient Cohort Information.

| **Characteristic** | **TCGA Cohort**  **(n = 258)** | | **Validation Cohort**  **(GSE39582, n = 585)** | |
| --- | --- | --- | --- | --- |
|  | No. | % | No. | % |
| **Status** | | | | |
| Alive | 219 | 84.89 | 385 | 65.81 |
| Dead | 39 | 15.11 | 194 | 33.16 |
| N/A | - | - | 6 | 1.03 |
| **Sex** | | | | |
| Male | 136 | 52.71 | 322 | 55.04 |
| Female | 122 | 47.29 | 263 | 44.96 |
| **Pathologic stage** | | | | |
| Stage 0 | - | - | 4 | 0.68 |
| Stage I&II | 139 | 53.88 | 309 | 52.82 |
| Stage III&IV | 119 | 46.12 | 270 | 46.15 |
| N/A | - | - | 2 | 0.34 |
| **Retrospective collection indicator** | | | | |
| No | 155 | 60.08 | - | - |
| Yes | 103 | 39.92 | - | - |
| **Race** | | | | |
| American indian or alaska native | 1 | 0.39 | - | - |
| Asian | 11 | 4.27 | - | - |
| Black or african american | 58 | 22.48 | - | - |
| White | 188 | 72.89 | - | - |
| **The year of initial** **pathologic diagnosis** | | | | |
| Median | 2010 | - | - | - |
| range | 1998 | - | - | - |
| **Age at initial pathologic diagnosis, years** | | | | |
| Median | 66 | - | 69 | - |
| Range | 31-90 | - | 22-97 | - |
| **Microsatellite status** | | | | |
| MicroSatellite stability (mss) | 165 | 63.95 | - | - |
| MicroSatellite Instability-High (msi-l) | 45 | 17.44 | - | - |
| MicroSatellite Instability-Low (msi-h) | 46 | 17.83 | - | - |
| Indeterminate | 2 | 0.78 | - | - |

## **Table S2.** Top 20 Most Enriched Functions of the 1063 Functional Genes

| **Function** | ***p*-value** |
| --- | --- |
| rRNA processing | 1.10×10^−11^ |
| negative regulation of transcription from RNA polymerase II promoter | 5.41×10^−11^ |
| positive regulation of transcription from RNA polymerase II promoter | 3.22×10^−10^ |
| positive regulation of transcription, DNA-templated | 4.61×10^−10^ |
| G1/S transition of mitotic cell cycle | 3.06×10^−9^ |
| canonical Wnt signaling pathway | 7.36×10^−9^ |
| positive regulation of telomere maintenance via telomerase | 1.01×10^−8^ |
| protein stabilization | 6.84×10^−8^ |
| viral process | 2.82×10^−7^ |
| positive regulation of canonical Wnt signaling pathway | 3.68×10^−7^ |
| anaphase-promoting complex-dependent catabolic process | 4.77×10^−7^ |
| positive regulation of telomerase RNA localization to Cajal body | 5.74×10^−7^ |
| transcription initiation from RNA polymerase II promoter | 6.71×10^−7^ |
| protein ubiquitination involved in ubiquitin-dependent protein catabolic process | 7.64×10^−7^ |
| G2/M transition of mitotic cell cycle | 1.15×10^−6^ |
| cell division | 1.20×10^−6^ |
| proteasome-mediated ubiquitin-dependent protein catabolic process | 2.36×10^−6^ |
| maturation of LSU-rRNA from tricistronic rRNA transcript (SSU-rRNA, 5.8S rRNA, LSU-rRNA) | 2.77×10^−6^ |
| regulation of ubiquitin-protein ligase activity involved in mitotic cell cycle | 2.89×10^−6^ |
| response to drug | 2.95×10^−6^ |

## **Table S3.** Top 20 Most Enriched KEGG Pathways of the 1063 Functional Genes

| **Function** | ***p*-value** |
| --- | --- |
| Pathways in cancer | 7.93×10^−15^ |
| Proteoglycans in cancer | 3.92×10^−14^ |
| Cell cycle | 1.70×10^−10^ |
| Hippo signaling pathway | 7.13×10^−10^ |
| Wnt signaling pathway | 5.00×10^−8^ |
| Glioma | 5.15×10^−8^ |
| HTLV-I infection | 1.44×10^−7^ |
| Prostate cancer | 1.51×10^−7^ |
| Melanogenesis | 4.58×10^−7^ |
| Progesterone-mediated oocyte maturation | 5.27×10^−7^ |
| Non-small cell lung cancer | 6.50×10^−7^ |
| Thyroid hormone signaling pathway | 1.62×10^−6^ |
| Oocyte meiosis | 2.42×10^−6^ |
| Basal cell carcinoma | 2.61×10^−6^ |
| Signaling pathways regulating pluripotency of stem cells | 8.39×10^−6^ |
| Ribosome biogenesis in eukaryotes | 8.43×10^−6^ |
| Bladder cancer | 4.42×10^−5^ |
| Small cell lung cancer | 7.61×10^−5^ |
| Adherens junction | 8.67×10^−5^ |
| Chronic myeloid leukemia | 1.04×10^−4^ |

## **Table S4.** Top 20 Most Enriched Functions of the 185 Core Functional Genes

| **Function** | ***p*-value** |
| --- | --- |
| rRNA processing | 1.20×10^−11^ |
| positive regulation of telomerase RNA localization to Cajal body | 7.69×10^−11^ |
| positive regulation of telomere maintenance via telomerase | 1.10×10^−9^ |
| positive regulation of protein localization to Cajal body | 8.08×10^−7^ |
| positive regulation of transcription from RNA polymerase II promoter | 1.38×10^−6^ |
| maturation of LSU-rRNA from tricistronic rRNA transcript (SSU-rRNA, 5.8S rRNA, LSU-rRNA) | 1.44×10^−6^ |
| positive regulation of establishment of protein localization to telomere | 1.44×10^−6^ |
| negative regulation of transcription from RNA polymerase II promoter | 2.11×10^−6^ |
| canonical Wnt signaling pathway | 2.62×10^−6^ |
| chromatin remodeling | 3.43×10^−6^ |
| negative regulation of transcription, DNA-templated | 5.69×10^−6^ |
| protein stabilization | 1.39×10^−5^ |
| cell division | 2.11×10^−5^ |
| viral process | 8.06×10^−5^ |
| histone H3 acetylation | 8.56×10^−5^ |
| regulation of ubiquitin-protein ligase activity involved in mitotic cell cycle | 9.03×10^−5^ |
| positive regulation of transcription, DNA-templated | 1.14×10^−4^ |
| ribosomal large subunit biogenesis | 1.27×10^−4^ |
| protein K11-linked ubiquitination | 1.73×10^−4^ |

## **Table S5.** Top 20 Enriched KEGG Pathways of the 185 Core Functional Genes

| **Function** | ***p*-value** |
| --- | --- |
| Ribosome biogenesis in eukaryotes | 6.95×10-^8^ |
| Cell cycle | 1.10×10^−4^ |
| HTLV-I infection | 1.34×10^−4^ |
| Wnt signaling pathway | 2.48×10^−4^ |
| Progesterone-mediated oocyte maturation | 3.50×10^−4^ |
| Melanogenesis | 8.14×10^−4^ |
| Thyroid hormone signaling pathway | 1.76×10^−3^ |
| Prostate cancer | 2.21×10^−3^ |
| Pathways in cancer | 2.57×10^−3^ |
| RNA transport | 4.76×10^−3^ |
| Ubiquitin mediated proteolysis | 4.93×10^−3^ |
| Oocyte meiosis | 6.39×10^−3^ |
| Hippo signaling pathway | 8.31×10^−3^ |
| Thyroid cancer | 9.60×10^−3^ |
| Basal cell carcinoma | 9.80×10^−3^ |
| Proteoglycans in cancer | 1.15×10^−2^ |
| Chronic myeloid leukemia | 2.42×10^−2^ |
| Viral carcinogenesis | 3.77×10^−2^ |
| Salivary secretion | 4.26×10^−2^ |
| Endometrial cancer | 4.51×10^−2^ |

## **Table S6.** Twelve Functional Genes Associated with the Prognosis of COAD

| **No.** | **Genes** | **Description** | **UNIVARIATE ANALYSIS** | **MULTIVARIATE ANALYSIS** |
| --- | --- | --- | --- | --- |
|  |  |  | **modality**  ***p-*value** | **modality**  ***p-*value** |
| 1 | *TPM2* | Tropomyosin 2 | 2.51×10^−3^ | 1.03×10^−2^ |
| 2 | *STMN2* | Stathmin 2 | 8.81×10^−3^ | 4.38×10^−2^ |
| 3 | *CHMP4C* | Charged Multivesicular Body Protein 4C | 1.24×10^−2^ | 4.31×10^−2^ |
| 4 | *WDR1* | WD Repeat Domain 1 | 1.34×10^−2^ | 4.40×10^−2^ |
| 5 | *CPT2* | Carnitine Palmitoyltransferase 2 | 1.77×10^−2^ | 1.42×10^−2^ |
| 6 | *DUSP14* | Dual Specificity Phosphatase 14 | 2.16×10^−2^ | 4.30×10^−2^ |
| 7 | *KDM1A* | Lysine Demethylase 1A | 2.59×10^−2^ | 2.16×10^−2^ |
| 8 | *NFE2L1* | Nuclear Factor, Erythroid 2 Like 1 | 3.96×10^−2^ | 7.17×10^−3^ |
| 9 | *TBL3* | Transducin Beta Like 3 | 3.97×10^−2^ | 6.39×10^−3^ |
| 10 | *GRIA3* | Glutamate Ionotropic Receptor AMPA Type Subunit 3 | 4.31×10^−2^ | 6.92×10^−3^ |
| 11 | *TGFBR3* | Transforming Growth Factor Beta Receptor 3 | 4.59×10^−2^ | 4.16×10^−2^ |
| 12 | *FGFR2* | Fibroblast Growth Factor Receptor 2 | 4.85×10^−2^ | 3.83×10^−2^ |

## **Table S7.** Thirteen Functional Edges Associated with the Prognosis of COAD

| **No.** | **Edges** | **Description** | **UNIVARIATE ANALYSIS** | **MULTIVARIATE ANALYSIS** |
| --- | --- | --- | --- | --- |
|  |  |  | **modality**  ***p-*value** | **modality**  ***p-*value** |
| 1 | *E2F1*-*KDM1A* | E2F Transcription Factor 1; Lysine Demethylase 1A | 7.51×10^−5^ | 2.04×10^−2^ |
| 2 | *ACTG2*-*SPTBN2* | Actin Gamma 2, Smooth Muscle; Spectrin Beta, Non-Erythrocytic 2 | 3.03×10^−3^- | 2.63×10^−2^ |
| 3 | *PPM1G*-*STMN2* | Protein Phosphatase, Mg2+/Mn2+ Dependent 1G; Stathmin 2 | 6.19×10^−3^ | 8.96×10^−3^ |
| 4 | *WDR3*-*PUS1* | WD Repeat Domain 3; Pseudouridine Synthase 1 | 1.99×10^−2^ | 4.55×10^−2^ |
| 5 | *PSMA6*-*SPTBN2* | Proteasome Subunit Alpha 6; Spectrin Beta, Non-Erythrocytic 2 | 2.44×10^−2^ | 3.41×10^−3^ |
| 6 | *UBE2S*-*RAD18* | Ubiquitin Conjugating Enzyme E2 S; RAD18 E3 Ubiquitin Protein Ligase | 3.32×10^−2^ | 2.81×10^−2^ |
| 7 | *ARRDC4*-*HECTD3* | Arrestin Domain Containing 4; HECT Domain E3 Ubiquitin Protein Ligase 3 | 3.37×10^−2^ | 1.01×10^−2^ |
| 8 | *HSP90AB1*-*CHEK1* | Heat Shock Protein 90 Alpha Family Class B Member 1; Checkpoint Kinase 1 | 3.43×10^−2^ | 2.58×10^−2^ |
| 9 | *ESR1*-*E2F1* | Estrogen Receptor 1; E2F Transcription Factor 1 | 3.46×10^−2^ | 2.41×10^−2^ |
| 10 | *SPTBN2*-*SPTAN1* | Spectrin Beta, Non-Erythrocytic 2; Spectrin Alpha, Non-Erythrocytic 1 | 3.97×10^−2^ | 3.02×10^−2^ |
| 11 | *CBX8*-*HOXA9* | Chromobox 8; Homeobox A9 | 4.26×10^−2^ | 1.66×10^−3^ |
| 12 | *SOX9*-*UBE2I* | SRY-Box 9; Ubiquitin Conjugating Enzyme E2 I | 4.38×10^−2^ | 3.75×10^−2^ |
| 13 | *BYSL*-*RRP12* | Bystin Like; Ribosomal RNA Processing 12 Homolog | 4.70×10^−2^ | 3.83×10^−2^ |

## Table S8. Eleven Genes Related to Colorectal Cancer

| **Genes** | **Description** | **Node degree** | **Related to CRC** | **In the five known cancer gene sets** |
| --- | --- | --- | --- | --- |
| *UBE2I* | Ubiquitin Conjugating Enzyme E2 I | 66 | YES | NO |
| *JUB* | Ajuba LIM Protein | 36 | YES | NO |
| *CCND1* | Cyclin D1 | 29 | YES | YES |
| *UBE2S* | Ubiquitin Conjugating Enzyme E2 S | 27 | YES | NO |
| *WNT2* | Wnt Family Member 2 | 22 | YES | YES |
| *CNTD2* | Cyclin N-Terminal Domain Containing 2 | 20 | YES | NO |
| *TRIB3* | Tribbles Pseudokinase 3 | 19 | YES | NO |
| *BOP1* | BOP1 Ribosomal Biogenesis Factor | 19 | YES | NO |
| *MET* | MET Proto-Oncogene, Receptor Tyrosine Kinase | 17 | YES | YES |
| *HDAC2* | Histone Deacetylase 2 | 15 | YES | YES |
| *GTPBP4* | GTP Binding Protein 4 | 15 | YES | NO |
